# Seriousness and outcomes of reported adverse drug reactions in old and new antiseizure medications: a pharmacovigilance study using EudraVigilance database

**DOI:** 10.3389/fphar.2024.1411134

**Published:** 2024-07-24

**Authors:** Michael Magdy Fahmy Girgis, Gergely Farkasinszky, Klára Fekete, István Fekete, Miklós Vecsernyés, Ildikó Bácskay, László Horváth

**Affiliations:** ^1^ Department of Pharmaceutical Surveillance and Economy, Faculty of Pharmacy, University of Debrecen, Debrecen, Hungary; ^2^ Division of Nuclear Medicine and Translational Imaging, Department of Medical Imaging, Faculty of Medicine, University of Debrecen, Debrecen, Hungary; ^3^ Department of Neurology, Faculty of Medicine, University of Debrecen, Debrecen, Hungary; ^4^ Healthcare Industry Institute, University of Debrecen, Debrecen, Hungary; ^5^ Department of Pharmaceutical Technology, Faculty of Pharmacy, University of Debrecen, Debrecen, Hungary

**Keywords:** seizure, antiseizure medications (ASMs), adverse events, adverse drug reaction, pharmacovgilance, Eudravigilance database, epilepsy, outcome

## Abstract

**Introduction:** Epilepsy is a widespread disease requiring long-term drug treatment. The aim of this study was to collect information on reported suspected adverse drug reactions (sADRs) of antiseizure medications (ASMs) and study their seriousness and outcomes in various system organ classifications (SOCs). We intended to compare old and new ASMs’ ADRs.

**Methods:** Using EudraVigilance (EV) database, we extracted line listings of reported sADRs with different ASMs over the period from January 2012 to December 2021. The list of ASMs was compiled according to the Anatomical therapeutic chemical classification system. The Medical Dictionary for Regulatory Activities version 24.0 was used for determining the SOCs of individual reported preferred terms (PTs) sADRs. In addition, we calculated the Reporting Odds Ratio (ROR), 95% confidence interval (95% CI), p-value (statistically significant if p< 0.05) and chi-square statistics.

**Results:** A total of 276,694 reports were contained in the exported line listings which included 1,051,142 individual sADRs reported as PTs such as seizure (3.49%), drug ineffective (2.46%), somnolence (1.32%), dizziness (1.29%) and represented four SOCs: nervous system disorders (19.26%), general disorders and administration site conditions (14.39%), psychiatric disorders (11.29%) and injury, poisoning and procedural complications (9.79). Among patients, the age group between 18 and 64 years had the highest percentage (52.40%), followed by those aged over 64 years (18.75%). Of all the reported PTs, 882,706 (83.98%) had reported seriousness. Old ASMs had a significant positive association with *“caused/prolonged hospitalisation”*, *“congenital anomaly”*, *“disabling”*, *“life threatening”* and *“results in death”*, while new ASMS with *‘other medically important condition’*. There were 386 (0.04%) PTs related to Sudden Unexpected Death in Epilepsy (SUDEP).

**Conclusion:** In our study, we examined 10 years’ reported sADRs of ASMs in the EV international database. The majority of PTs were serious. Old ASMs were generally more commonly associated with undesired outcomes and seriousness. Considering their expected seriousness and outcomes, the safety profile of the different ASMs, can play a cardinal role in the selection of ASMs.

## Introduction

Epilepsy is widely spread all over the world with millions suffering from it and its considerable morbidity and mortality (Beghi et al., 2020). Looking over the trends in age-specific incidence over the last decades, epilepsy decreased in the youngest age groups due to improvements in perinatal care, better sanitation, and increased control of infectious diseases (Collaborators, 2019). In contrast, it increased in the elderly, possibly due to improved life expectancy accompanied by increased epileptogenic conditions such as stroke, tumours, and neurodegenerative disorders (Marsh and Rao, 2002). Epilepsy usually requires lifelong treatment, even after epilepsy surgery. Antiseizure medications (ASMs) are important for most people with epilepsy (PWE).

ASMs are classified as old and new types. Although many new types of ASMs were made available, no considerable improvement in tolerability and efficacy were proved (Löscher and Schmidt, 2011), thus many old type ASMs are still widely used (Vajda and Eadie, 2014). Although this provided more therapeutic options (Beyenburg et al., 2010), adverse drug reactions, interactions or hypersensitivity reactions were not eliminated (Elger and Schmidt, 2008).

According to the European Medicines Agency (EMA), adverse drug reaction (ADR) is a response to a medicinal product which is harmful and unintended. Response in this context means that a causal relationship between the medicinal product and an adverse event is at least a reasonable possibility (Agency, 2017), which contribute outstandingly to the debilitation of health quality and treatment failure in PWE (Perucca and Gilliam, 2012). Many efforts were exerted to quantify ASMs drug toxicity and lower their burden.

According to the World Health Organization, pharmacovigilance (PV) can be defined as “the science and activities relating to the detection, assessment, understanding and prevention of the adverse effects of drugs or any other possible drug-related problems” (Team WHOQAaSoM, 2002). PV reinforces the safe and appropriate use of drugs and faces the considerable problem of increased ADRs due to increasing reporting rates and contribution of useful interventions (Campbell et al., 2014). The past decades have witnessed marked development in drug safety measurement due to large PV databases such as VigiBase and EudraVigilance (EV), and automation of statistical methods and safety signal detection (Bihan et al., 2020). These PV databases are valuable sources of information that can be implied in numerous retrospective observational studies. The EV database - first operated in 2001- has substantial importance with regard to the management of suspected ADRs (sADRs) reports and safety evaluation of authorized medicines in the European Economic Area (EEA) (Postigo et al., 2018). Marketing authorisation holders (MAHs) and sponsors of clinical trials have obligations to report sADRs during phases of medicinal products development and after getting authorized in the EEA. EV supports the electronic exchange of sADRs using Individual Case Safety Reports (ICSRs) between the EMA, National Competent Authorities, MAHs and sponsors of interventional clinical trials and non-interventional studies in the EEA, besides its contribution to early detection of possible safety signals, monitoring and evaluation of potential safety issues and decision making process in the frame of EU Risk Management Strategy (Note for guidance, 2010; EudraVigilance, 2023).

The aim of this study was to address the reported sADRs over a 10-year period and the possible trends change using EV seriousness and System Organ Classes (SOC) of sADRs and their outcomes. Furthermore, we aimed to find possible associations between ASMs and occurrences of sADRs having certain outcomes, seriousness or SOC. Studying the possible association between ASMs and occurrence of sudden unexpected death in epilepsy (SUDEP) was an outstanding goal. Besides, we were interested in comparing old and new type ASMs.

## Methods

### Data source of adverse drug reactions

The EV system was used to retrieve information on sADRs, based on ICSRs. In accordance with Regulation (EU) 2016/679, the General Data Protection Regulation, and Regulation (EU) 2018/1725, the EU Data Protection Legislation, European database of suspected adverse drug reaction reports (www.adrreports.eu) was used as an access tool (Krzysztofek, 2017; Office, 2019; Ruggiero et al., 2022). Line listing functionality was applied to export results of reported sADRs with different ASMs (which were considered at the level of chemical structure or active pharmaceutical ingredient) over the period from January 2012 to December 2021 in a tabulated format for further analyses. Line listings were extracted.

### Adverse drug reactions

The Medical Dictionary for Regulatory Activities (MedDRA) version 24.0 was used for determining the SOCs of individual reported preferred terms (PTs) for each sADR.

According to EV, seriousness is classified as *“other medically important condition”*, *“caused/prolonged hospitalisation”*, *‘congenital anomaly’*, *“disabling”*, *“life threatening”*, and *“results in death”*; outcome is classified as *“fatal”*, *“not recovered/not resolved”*, *“recovered/resolved”*, *“recovered/resolved with sequelae”*, and *“recovering/resolving”* (Note for guidance, 2010).

### Antiseizure medications

List of ASMs (or otherwise known as antiepileptics) was compiled according to Anatomical Therapeutic Chemical (ATC) classification system N03A subgroups (WHOCC, 2016). Chemical grouping of ATC was employed for aggregate analysis.

Considering to use the term established or old, but nowadays, established ASMs include new ASMs already besides the old ones. So, established may differ between countries, therefore we decided to name the groups old and new ASMs. Old ASMs refer to those which were put on the market before 1990 and belong to first and second generation ASMs. Old and new types of ASMs were considered as follows (their common abbreviations are shown in round brackets):

#### Old types

Phenobarbital (PB), Barbexaclone, Metharbital, Methylphenobarbital, Primidone (PRM), Clonazepam (CZP), Clorazepate potassium, Carbamazepine (CBZ), Aminobutyric acid, Valproic acid and Sodium Valproate (VPA), Ethotoin, Mephenytoin, Phenytoin (PHT), Beclamide, Phenacemide, Pheneturide, Sultiame (SUL), Paramethadione, Trimethadione, Ethosuximide (ESM), Mesuximide, Phensuximide.

#### New types

Clobazam (CLB), Eslicarbazepine (ESL), Oxcarbazepine (OXC), Rufinamide (RUF), Tiagabine (TGB), Vigabatrin (VGB), Fosphenytoin, Brivaracetam (BRV), Cenobamate (CNB), Felbamate (FBM), Fenfluramine (FEN), Gabapentin (GBP), Lacosamide (LCM), Lamotrigine (LTG), Levetiracetam (LEV), Perampanel (PER), Pregabalin (PGB), Retigabine (RTG), Stiripentol (STP), Topiramate (TPR), Zonisamide (ZNS).

##### Statistical analysis

Data arrangement and analysis was done using Microsoft Office Excel 2019 and SPSS for Windows 26.0 (SPSS Inc. Chicago, USA). ROR with its 95% confidence interval (95%CI), proportional reporting ratio (PRR), p-value and chi-square statistic were calculated for different ASMs for analysing outcomes and seriousness. Differences were considered significant if p< 0.05. The number of reported sADRs were pooled into a two by two contingency tables correlating certain ASM or ASM pharmacological group, and those with other ASMs or ASMs pharmacological groups, to the various outcomes, seriousness criteria and SUDEP in order to calculate ROR, PRR and chi-square.

ROR allows the estimation of relative risk and removal of biases in pharmacovigilance (Rothman et al., 2004).

According to the EMA, signal of disproportionate reporting can show the association between drug-event pair in the database which can be generated from spontaneous adverse drug reaction reporting systems based on the following criteria (Note for guidance, 2010).

Fulfilling the following criteria for generating a signal according to EudraVigilance (Agency, 2016):

a) When the PRR is displayed with its 95% confidence interval:
- the lower bound of the 95% confidence interval is greater than or equal to one
- the number of individual cases is greater than or equal to three
b) When the PRR is displayed with χ2 statistic:
- the PRR > 2- χ2> four

PRR can be used as a direct measure of the strength of the signal and it can also be used to determine unexpectedness relative to the background of the rest of the database. ROR provides additional information over PRR, which can be important in evaluating the link between ADRs and drugs (Evans et al., 2001).

## Results

### Reports overview

The exported line listings consisted of a total of 276,694 reports (From January 2012 to December 2021). It included 1,051,142 individual sADRs reported as PTs. Overall, the EV database held a total of 22,301,140 sADRs reports by 31st of December 2021 (EMA, 2022), so our exported ASMs line listings constituted 1.24% of the database. As for gender, 106,834 (38.61%) of the reports included information about males, whereas 148,957 (53.83%) were from females and the remaining reports had no specified gender (20,903; 7.56%). The majority of the reports included the age group between 18 and 64 years, followed by those in the group over 65 years, while children aged 2 years or under had the lowest percentage (Table 1). Of all the reports, 199,956 (72.27%) were made by healthcare professional. Fewer reports came from the EEA than non-EEA countries (99,243; 35.87% vs. 177,271; 64.07%). Regarding the ASM groups (according to ATC classification system), the group of other ASMs constituted the vast majority of the reports (167,065; 60.38%), followed by fatty acid derivatives (41,733; 15.08%) and carboxamides (32,295; 11.67%). PGB (57,497; 20.78%), VPA (35,235; 12.73%), LEV (29,146; 10.53%), CBZ (23,294; 8.42%) and LTG (22,835; 8.25%) were the five ASMs with the highest numbers of sADRs reported. Detailed information is listed in Table 1. There were more reports of new ASMs than old ones (Table 2) especially for women, which was statistically significant χ^2^ (2, N = 1,051,144) = 11,356.9014, *p* < 0.00001. Calculating the average number of PT (number of PTs/number of ASMs) for old and new ASMs, the results were 16,202.7 vs 33,924.1, respectively. If the average PTs per reports is taken into consideration, it was found to be 3.72 for newer ASMs and 3.96 for old ASMs.

**TABLE 1:** Reports overview of antiseizure medications in EudraVigilance database between 2012 and 2021.

	Number of reports in EudraVigilance (%)
Total number of reports	276,694 (100)
Age groups	
0–2 years	8,977 (3.24)
3–17 years	24,789 (8.96)
18–64 years	131,984 (47.7)
>64	50,291 (18.18)
Not specified	60,653 (21.92)
ASMs (approval in the EU/USA)	
*Barbiturates*	5,569 (2.01)
Phenobarbital (1912/1912)	4,607 (1.67)
Barbexaclone (1983/1983)	24 (0.01)
Metharbital (1952/1952)	1 (0)
Methylphenobarbital (1932/1932)	172 (0.06)
Primidone (1960/1954)	765 (0.28)
*Benzodiazepines*	18,931 (6.84)
Clobazam (1975/2011)	4,245 (1.53)
Clonazepam (1968/1976)	14,679 (5.31)
Clorazepate potassium (1972/1972)	7 (0)
*Carboxamides*	32,295 (11.67)
Carbamazepine (1965/1968)	23,294 (8.42)
Eslicarbazepine (2010/2013)	1,980 (0.72)
Oxcarbazepine (1990/2000)	6,678 (2.41)
Rufinamide (2007/2009)	343 (0.12)
*Fatty Acid derivatives*	41,733 (15.08)
Aminobutyric acid (undetermined)	19 (0.01)
Tiagabine (1996/1990)	308 (0.11)
Valproic acid and Sodium Valproate (1970/1978)	35,235 (12.73)
Vigabatrin (1989/2009)	6,171 (2.23)
*Hydantoins*	10,467 (3.78)
Ethotoin (1957/1957)	4 (0)
Fosphenytoin (1998/1996)	713 (0.26)
Mephenytoin (1947/1947)	11 (0)
Phenytoin (1939/1953)	9,739 (3.52)
*Other Antiepileptics*	167,065 (60.38)
Beclamide (undetermined/1952)	2 (0)
Brivaracetam (2016/2015)	2,944 (1.06)
Cannabidiol (2019/2018)	4,722 (1.71)
Cenobamate (2019/2019)	389 (0.14)
Felbamate (1994/1993)	189 (0.07)
Fenfluramine (2020/2020)	309 (0.11)
Gabapentin (1994/1994)	19,369 (7)
Lacosamide (2008/2009)	10,542 (3.81)
Lamotrigine (1991/1994)	22,835 (8.25)
Levetiracetam (2000/1999)	29,146 (10.53)
Perampanel (2012/2012)	2,413 (0.87)
Phenacemide (1951/1951)	2 (0)
Pheneturide (1951/1951)	2 (0)
Pregabalin (2005/2005)	57,497 (20.78)
Retigabine (2011/2011)	463 (0.17)
Stiripentol (2007/2018)	614 (0.22)
Sultiame (1960/not approved)	170 (0.06)
Topiramate (1995/1996)	13,214 (4.78)
Zonisamide (2007/2000)	2243 (0.81)
*Oxazolidines*	10 (0)
Paramethadione (1949/1949)	1 (0)
Trimethadione (1946/1946)	9 (0)
*Succinimides*	624 (0.23)
Ethosuximide (1958/1960)	580 (0.21)
Mesuximide (1957/1957)	43 (0.02)
Phensuximide (1953/1953)	1 (0)

**TABLE 2:** Number of suspected adverse drug reactions of old and new antiseizure medications by gender and year reported.

	Number of sADRs (in % of total sADRs)	
	*Old ASMs (%)*	*New ASMs (%)*
Total	372,660 (35.45)	678,482 (64.55)
Gender		
Male	162,330 (15.44)	228,844 (21.77)
Female	188,410 (17.92)	415,526 (39.53)
Not Specified	21,922 (2.09)	34,112 (3.25)
Year		
2012	27,489 (2.62)	59,165 (5.63)
2013	36,095 (3.43)	56,517 (5.38)
2014	37,793 (3.6)	56,487 (5.37)
2015	31,134 (2.96)	58,445 (5.56)
2016	27,089 (2.58)	55,144 (5.25)
2017	46,758 (4.45)	73,937 (7.03)
2018	29,981 (2.85)	65,693 (6.25)
2019	40,368 (3.84)	79,795 (7.59)
2020	42,226 (4.02)	76,339 (7.26)
2021	53,729 (5.11)	96,960 (9.22)

sADRs: suspected adverse drug reactions; ASM: antiseizure medications.

### Reported PTs

The ten most frequently reported PTs were seizure (36,694; 3.49%), drug ineffective (25,873; 2.46%), somnolence (13,903; 1.32%), dizziness (13,562; 1.29%), off label use (11,953; 1.14%), rash (10,877; 1.03%), pain (10,327; 0.98%), fatigue (10,021; 0.95%), toxicity to various agents (9,773; 0.93%) and drug interaction (9,726; 0.93%). In males, the ten most frequently reported PTs were seizure (14,602; 1.39%), drug ineffective (9,106; 0.87%), somnolence (5,117; 0.49%), off label use (4,585; 0.44%), dizziness (4,033; 0.38%), drug interaction (4,006; 0.38%), rash (3,685; 0.35%), epilepsy (3,635; 0.35%), toxicity to various agents (3,457; 0.33%) and pyrexia (3,211; 0.31%). In females, the ten most frequently reported PTs were seizure (16,168; 1.54%), drug ineffective (13,459; 1.28%), dizziness (8,239; 0.78%), somnolence (7,534; 0.72%), pain (6,793; 0.65%), off label use (6,255; 0.60%), rash (6,078; 0.58%), fatigue (5,968; 0.57%), nausea (5,918; 0.56%) and headache (5,765; 0.55%).

**FIGURE 1:**
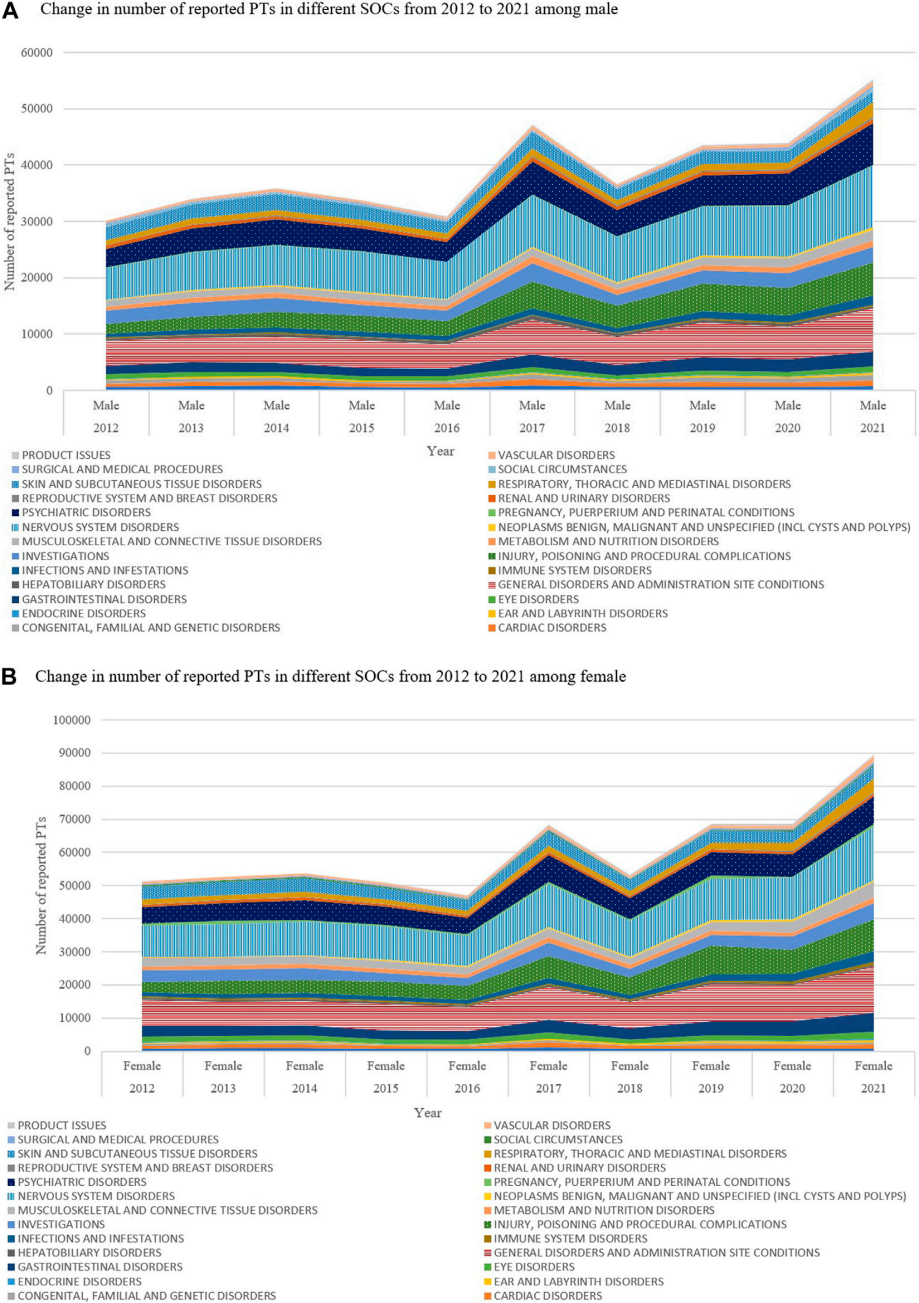
**(A)** Change in number of reported PTs in different SOCs from 2012 to 2021 among male **(B)** Change in number of reported PTs in different SOCs from 2012 to 2021 among female.

**FIGURE 2:**
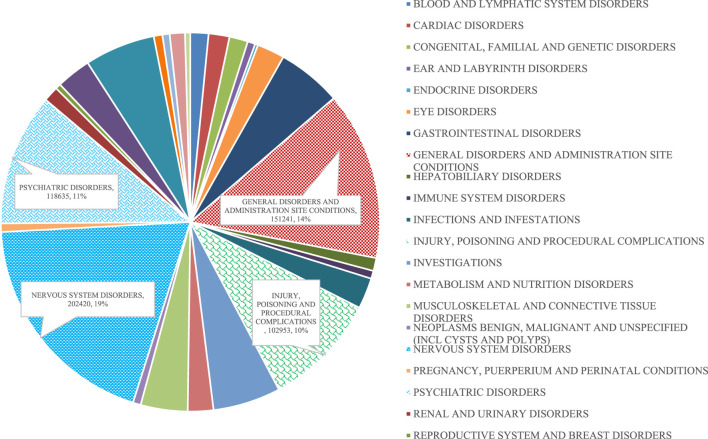
Proportions of different SOCs.

**FIGURE 3:**
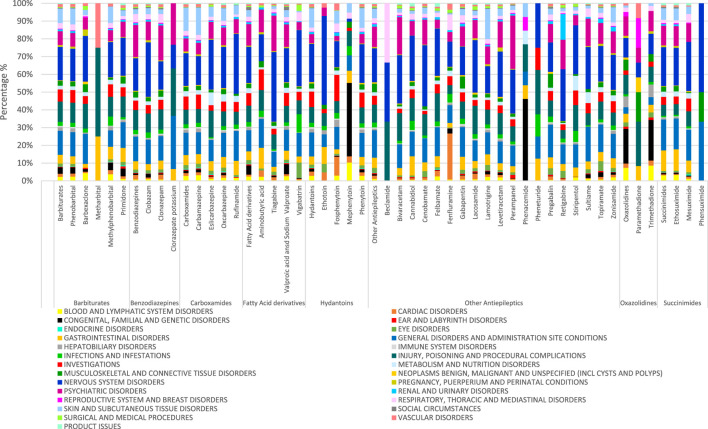
Proportions of SOCs by different ASMs.

The reported PTs for the different SOCs between 2012 and 2021 are provided in Figure 1. Looking at the total number of reported PTs, the highest was recorded in 2021 (150,689; 14.34%), followed by 2017, 2019 and 2020 (120,695; 11.48%, 120,163; 11.43% and 118,564; 11.28%, respectively). The lowest number of total reported PTs (82,232; 7.82%) was recorded in 2016. In a similar way to what was noticed concerning the total number of PTs, both males and females had the highest number of reported PTs in 2021 (55,245; 5.26% and 89,625; 8.53%, respectively). In all years, the most frequently reported sADRs belonged to four SOCs: *‘nervous system disorders’* (20,2420; 19.26%), *‘general disorders and administration site conditions’* (151,240; 14.39%), *‘psychiatric disorders’* (118,635, 11.29%) and *‘injury, poisoning and procedural complications’* (102,953; 9.79%) (Table 3; Figure 2). However, the lowest number of PTs was in the SOC of endocrine disorders in all years except in 2012 and 2013, when the SOC of product issues had the lowest frequency. Figure 3 shows the proportions of SOCs in different ASMs. Similarly, in both males and females, the highest number of PTs belonged to the SOC of *‘nervous system disorders’*, followed by the SOC of *‘general disorders and administration site conditions’*. Comparing the old and new ASMs pronounced unfavourable effect can be seen in the case of *‘blood and lymphatic system disorders’*, *‘congenital, familial and genetic disorders’*, and *‘hepatobiliary disorders’* (Table 3).

**TABLE 3:** Number of PTs by SOC and numbers of PTs related to old and new ASMs with their Reporting Odds Ratio.

SOC	Number of PTs (%)	Number of old ASMs (%)	Number of new ASMs (%)	Reporting odds ratio with its 95% confident interval and p-value
Blood and lymphatic system disorders	16,214 (1.54)	8,676 (0.83)	7,538 (0.72)	ROR = 2.12, 95% CI [2.09, 2.15] p< 0.0001
Cardiac disorders	18,707 (1.78)	6,253 (0.59)	12,454 (1.18)	ROR = 0.91, 95% CI [0.88, 0.94] p< 0.0001
Congenital, familial and genetic disorders	16,644 (1.58)	11,069 (1.05)	5,575 (0.53)	ROR = 3.69, 95% CI [3.66, 3.73] p< 0.0001
Ear and labyrinth disorders	6,795 (0.65)	2,282 (0.22)	4,513 (0.43)	ROR = 0.92, 95% CI [0.87, 0.97] p< 0.0001
Endocrine disorders	2,680 (0.25)	1,255 (0.12)	1,425 (0.14)	ROR = 1.61, 95% CI [1.53, 1.68] p< 0.0001
Eye disorders	247,86 (2.36)	6,089 (0.58)	18,697 (1.78)	ROR = 0.59, 95% CI [0.56, 0.62] p< 0.0001
Gastrointestinal disorders	57,747 (5.49)	19,827 (1.89)	37,920 (3.61)	ROR = 0.95, 95% CI [0.93, 0.97] p< 0.0001
General disorders and administration site conditions	151,241 (14.39)	47,355 (4.51)	103,885 (9.88)	ROR = 0.81, 95% CI [0.79, 0.82] p< 0.0001
Hepatobiliary disorders	11,601 (1.1)	6,111 (0.58)	5,490 (0.52)	ROR = 2.04, 95% CI [2.01, 2.08] p< 0.0001
Immune system disorders	7,075 (0.67)	2,261 (0.22)	4,814 (0.46)	ROR = 0.85, 95% CI [0.8, 0.9] p< 0.0001
Infections and infestations	27,773 (2.64)	8,579 (0.82)	19,194 (1.83)	ROR = 0.81, 95% CI [0.78, 0.84] p< 0.0001
Injury, poisoning and procedural complications	102,953 (9.79)	35,582 (3.39)	67,371 (6.41)	ROR = 0.96, 95% CI [0.94, 0.97] p< 0.0001
Investigations	60,734 (5.78)	26,551 (2.53)	34,183 (3.25)	ROR = 1.45, 95% CI [1.43, 1.46] p< 0.0001
Metabolism and nutrition disorders	22,989 (2.19)	8,832 (0.84)	14,157 (1.35)	ROR = 1.14, 95% CI [1.11, 1.17] p< 0.0001
Musculoskeletal and connective tissue disorders	42,077 (4)	10,419 (0.99)	31,658 (3.01)	ROR = 0.59, 95% CI [0.57, 0.61] p< 0.0001
Neoplasms benign, malignant and unspecified (incl. cysts and polyps)	7,044 (0.67)	1,713 (0.16)	5,331 (0.51)	ROR = 0.58, 95% CI [0.53, 0.64] p< 0.0001
Nervous system disorders	202,420 (19.26)	68,629 (6.53)	133,791 (12.73)	ROR = 0.92, 95% CI [0.91, 0.93] p< 0.0001
Pregnancy, puerperium and perinatal conditions	7,677 (0.73)	2,669 (0.25)	5,008 (0.48)	ROR = 0.97, 95% CI [0.92, 1.02] n.s
Psychiatric disorders	118,635 (11.29)	42,193 (4.01)	76,442 (7.27)	ROR = 1.01, 95% CI [0.99, 1.02] n.s
Renal and urinary disorders	13,630 (1.3)	4,058 (0.39)	9,572 (0.91)	ROR = 0.77, 95% CI [0.73, 0.81] p< 0.0001
Reproductive system and breast disorders	4,821 (0.46)	1,748 (0.17)	3,073 (0.29)	ROR = 1.04, 95% CI [0.98, 1.09] n.s
Respiratory, thoracic and mediastinal disorders	31,058 (2.95)	11,299 (1.07)	19,759 (1.88)	ROR = 1.04, 95% CI [1.02, 1.07] p< 0.0001
Skin and subcutaneous tissue disorders	63,215 (6.01)	27,102 (2.58)	36,113 (3.44)	ROR = 1.4, 95% CI [1.38, 1.41] p< 0.0001
Social circumstances	7,760 (0.74)	3,607 (0.34)	4,152 (0.39)	ROR = 1.59, 95% CI [1.54, 1.63] p< 0.0001
Surgical and medical procedures	6,478 (0.62)	1,535 (0.15)	4,943 (0.47)	ROR = 0.56, 95% CI [0.51, 0.62] p< 0.0001
Vascular disorders	13,575 (1.29)	4,994 (0.48)	8,581 (0.82)	ROR = 1.06, 95% CI [1.03, 1.1] p< 0.0001
Product issues	4,813 (0.46)	1,970 (0.19)	2,843 (0.27)	ROR = 1.26, 95% CI [1.21, 1.32] p< 0.0001
Not Specified	2 (0)	2 (0)	0 (0)	N/C

SOC: system organ classes; PT: preferred terms; ASM: antiseizure medication; ROR: reporting odds ratio; CI: confidence interval; n. s.: not significant; N/C: not countable.

As for **barbiturates**, there was a significant positive association with occurrences of PTs that belong to SOCs of ‘*blood and lymphatic system disorders’, ‘congenital, familial and genetic disorders’, and ‘hepatobiliary disorders’.*

In **benzodiazepines**, a significant positive association with occurrences of PTs was noticed in SOCs of *‘congenital, familial and genetic disorders’*, *‘pregnancy, puerperium and perinatal conditions’*.

With respect to **carboxamides**, a significant positive association with occurrences of PTs was noticed in SOCs of *‘blood and lymphatic system disorders’*, *‘endocrine disorders’*, *‘skin and subcutaneous tissue disorders’*, *‘pregnancy, puerperium and perinatal conditions’*.

ESL had the least negative effect on SOC. For example, in the case of CBZ the term *‘hepatobiliary disorders’* is common, in the case of OXC it is half and ESL is a quarter, ESL having the best ADR profile.

Regarding **fatty acid derivatives**, there was a significant positive association with occurrences of PTs that belong to *‘congenital, familial and genetic disorders’* (three-fold in males), *‘social circumstances’*.

With respect to **hydantoins**, a significant positive association with occurrences of PTs was noticed in SOCs of *‘cardiac disorders’*, *‘hepatobiliary disorders’* and *‘blood and lymphatic system disorders’.*

As for **other antiepileptics**, none was notable.

Regarding **oxazolidines**, *‘blood and lymphatic system disorders’*, *‘congenital, familial and genetic disorders’* had a significant positive association generally (same in males) and no association in females.

With respect to **succinimides**, there was a significant positive association with occurrences of PTs that belong to *‘blood and lymphatic system disorders’*, *‘gastrointestinal disorders’*.

### Seriousness

Of all the reported PTs, 882,706 (83.98%) had seriousness reported, of which 621,642 PTs (70.42%) were reported by healthcare professionals. The age group of 18–64 years had the highest number of PTs in all seriousness (Table 4) with only one exception of *‘congenital anomaly’* seriousness in which the patients in the group aged 2–17 years had the highest PTs number (11,715; 27.05%) followed by neonates under the age of 2 years (5,977; 13.80%).

**TABLE 4:** Number of reported suspected adverse drug reactions (sADRs) in different seriousness criteria by age groups.

Age group	Seriousness criteria (number of sADRs)							
	Other medically important condition	Caused/Prolonged hospitalisation	Congenital anomaly	Disabling	Life threatening	Results in death	Not specified/unknown	All
*0–2 years*	15,314	11,599	5,977	997	1,210	1,830	2,133	39,060
*3–17 years*	50,670	37,376	11,715	2,766	5,838	3,366	12,490	124,221
*18–64 years*	330,296	204,658	5,378	21,256	29,574	34,261	85,933	711,356
*65 years or older*	113,041	82,848	130	5,697	8,671	12,980	34,490	257,857
*Not Specified*	118,476	29,984	20,114	5,897	2,888	6,920	33,388	217,667
*All*	627,797	366,465	43,314	36,613	48,181	59,357	168,434	1,350,161

The most frequently reported seriousness criteria were *‘other medically important condition’* (632,691 PTs; 60.19%) followed by *‘caused/prolonged hospitalisation’* (371,185 PTs; 35.31%). The same trends were found in both males (226,702 PTs; 57.95% and 148,947 PTs; 38.08%, respectively) and females (371,775 PTs; 61.56% and 212,625 PTs; 35.21%, respectively). Of all the reported PTs, 5.79% had the seriousness criterion of *‘results in death’* (7.20% and 4.87% in males and females respectively).

In the chemical subgroups of **barbiturates**, in all seriousness criteria, there was a significant positive association except *‘other medically important condition’* and *‘disabling’,* but these two had negative associations. Among the barbiturates, mephenytoin can be highlighted in the *‘life threatening’* and *‘congenital anomaly’* criteria (Table 5). Methylphenobarbital had the highest OR of *‘results in death’* criterion in the group.

**TABLE 5:** A and B Associations of seriousness criteria by ASM chemical subgroups. A Seriousness criterion with positive association of ASM chemical subgroups. B Seriousness criterion with negative association of ASM chemical subgroups.

Criterion	ASM chemical subgroup	ROR	Lower 95%CI	Upper 95%CI	p-value	PRR	Lower 95%CI	Chi-square value
Caused/Prolonged Hospitalisation	Barbiturates	1.43	1.41	1.46	<0.001	1.24	1.23	700.24
	Benzodiazepines	1.24	1.23	1.26	<0.001	1.14	1.14	856.54
	Carboxamides	1.57	1.55	1.58	<0.001	1.31	1.31	5,229.80
	Hydantoins	1.59	1.57	1.61	<0.001	1.32	1.31	2,071.67
Congenital Anomaly	Barbiturates	1.69	1.64	1.74	<0.001	1.64	1.60	386.55
	Fatty Acid derivatives	11.45	11.43	11.47	<0.001	9.59	9.57	83,040.01
	Oxazolidines	24.06	23.62	24.49	<0.001	12.25	12.04	452.35
Disabling	Benzodiazepines	1.11	1.08	1.15	<0.001	1.11	1.07	33.19
	Fatty Acid derivatives	1.49	1.46	1.51	<0.001	1.46	1.44	901.91
Life Threatening	Barbiturates	1.51	1.45	1.56	<0.001	1.47	1.42	235.70
	Benzodiazepines	1.65	1.62	1.68	<0.001	1.60	1.58	1,251.79
	Carboxamides	1.42	1.39	1.44	<0.001	1.39	1.37	730.39
	Hydantoins	1.55	1.51	1.59	<0.001	1.51	1.48	481.40
Other Medically Important Condition	Hydantoins	1.34	1.32	1.36	<0.001	1.11	1.11	751.15
	Other Antiepileptics	1.19	1.18	1.20	<0.001	1.07	1.07	1,835.28
Results in Death	Barbiturates	2.11	2.07	2.15	<0.001	1.98	1.95	1,249.83
	Benzodiazepines	1.99	1.97	2.01	<0.001	1.89	1.87	3,246.13
	Hydantoins	1.74	1.71	1.78	<0.001	1.67	1.64	1,017.93
	Oxazolidines	5.25	4.74	5.75	<0.001	4.21	3.83	51.97

Criterion	ASM chemical subgroup	ROR	Lower 95%CI	Upper 95%CI	p-value	PRR	Lower 95%CI	Chi-square value
Caused/Prolonged Hospitalisation	Fatty Acid derivatives	0.90	0.89	0.91	<0.001	0.93	0.92	346.95
	Other Antiepileptics	0.73	0.73	0.74	<0.001	0.82	0.82	5,547.76
	Succinimides	0.80	0.70	0.89	<0.001	0.86	0.79	20.98
Congenital Anomaly	Benzodiazepines	0.43	0.38	0.48	<0.001	0.44	0.39	1,156.42
	Carboxamides	0.57	0.53	0.60	<0.001	0.58	0.54	910.72
	Hydantoins	0.46	0.39	0.53	<0.001	0.47	0.40	497.05
	Other Antiepileptics	0.17	0.15	0.20	<0.001	0.19	0.16	27,829.44
Disabling	Barbiturates	0.69	0.61	0.78	<0.001	0.70	0.62	72.42
	Carboxamides	0.55	0.51	0.60	<0.001	0.56	0.52	806.86
	Other Antiepileptics	0.97	0.94	0.99	<0.001	0.97	0.95	10.28
Life Threatening	Other Antiepileptics	0.64	0.62	0.65	<0.001	0.65	0.63	2,428.99
Other Medically Important Condition	Barbiturates	0.82	0.80	0.85	<0.001	0.92	0.91	205.15
	Carboxamides	0.85	0.84	0.86	<0.001	0.93	0.93	702.09
	Fatty Acid derivatives	0.79	0.78	0.80	<0.001	0.90	0.90	1,875.13
	Succinimides	0.77	0.68	0.86	<0.001	0.89	0.85	32.94
Results in Death	Carboxamides	0.82	0.80	0.85	<0.001	0.83	0.81	187.29
	Fatty Acid derivatives	0.82	0.80	0.85	<0.001	0.83	0.81	238.79
	Other Antiepileptics	0.75	0.74	0.77	<0.001	0.77	0.75	1,132.85

In terms of **benzodiazepines**, there was a significant positive association with *‘caused/prolonged hospitalisation’, ‘life threatening’* and *‘results in death’* seriousness criteria, while a significant negative association was found only with *‘congenital anomaly’* seriousness criterion. As for *‘other medically important condition’* seriousness criterion, there was no significant association in general. Clorazepate potassium can be highlighted since the strikingly high positive associations.

**Carboxamides** showed a significant positive association only with *‘caused/prolonged hospitalisation’* and *‘life-threatening’* seriousness criteria. However, a significant negative association was recognized with *‘results in death’*, *‘congenital anomaly’*, *‘disabling’* and *‘other’.*

It is worth mentioning that the highest ROR has been calculated in *‘congenital anomaly’* criterion by **fatty acid derivatives**, besides this *‘disabling’* had a significantly increased association. However, with seriousness criteria of *‘results in death’*, *‘caused/prolonged hospitalisation’* and *‘other medically important condition’*, there was a significant negative association. There was a significant positive association in VGB with *‘results in death’* criterion. There was a significant positive association in VPA with *‘congenital anomaly’*.

With respect to **hydantoins**, there was a significant positive association with *‘other medically important condition’*, *‘caused/prolonged hospitalisation’*, *‘life threatening’* and *‘results in death’* seriousness criteria, while a significant negative association was found with *‘congenital anomaly’* seriousness criterion in general. Having had a closer look at the ASMs, mephenytoin showed 26-fold higher reported odds for *‘congenital anomaly’*. There was a significant, three-fold and five-fold positive association of fosphenytoin with *‘results in death’* and *‘life threatening’* criteria, respectively.

Regarding **other antiepileptics**, a significant negative association was found with seriousness criteria of *‘congenital anomaly’* (TPM) and *‘life threatening’* (LTG, and ZNS). None of the positive associations could be detected in the five serious medical conditions among the criteria of seriousness, except in PGB, RGB, GBP and TPM detailed in *‘other medically important condition’*.

As for **oxazolidines**, there was a significant positive association with *‘results in death’* seriousness criterion, the reported odds being five times higher. Regarding *‘congenital anomaly’*, there was a 26-fold positive association in general and, a 34-fold association with trimethadione.

Regarding **succinimides**, there was a significant negative association with the seriousness criteria of *‘other medically important condition’* and *‘caused/prolonged hospitalisation’*.

Examining the cases labelled as *‘results in death’* (Supplementary Table S1), the most frequently reported PTs were listed for the following ASMs: PGB (8,407; 13.81%), GBP (8,316; 13.66%) and CLZ (7,877; 12.94%). Among the PTs with *‘caused/prolonged hospitalization’*, PGB (66,041; 17.79%), VPA (44,908; 12.10%), CBZ (42,313; 11.40%), LTG (42,300; 11.39%) and GBP (31,626; 8.52%) were the most common. Regarding *‘congenital anomaly’*, ASMs having the highest scores of reported PTs were VPA (27,330; 62.12%), LTG (3,082; 7.01%), TPM (2,634; 5.99%), CBZ (2,542; 5.78%) and LEV (2,211; 5.03%). Regarding *‘disabling’* criteria, the most frequently reported PTs included PGB (9,832; 26.62%), VPA (7,267; 19.68%), GBP (3,756; 10.17%), LTG (3,062; 8.29%) and CLZ (2,893; 7.83%). In case of *‘life-threatening’* term, the most frequently reported PTs were listed by patients taking VPA (6,828; 13.85%), PGB (6,477; 13.14%), LTG (6,334; 12.85%), CBZ (5,897; 11.97%) and CLZ (5,438; 11.03%).

Old ASMs (Supplementary Table S2), had a significant positive association with *‘caused/prolonged hospitalisation’* (ROR = 1.32, 95%CI: 1.31-1.32, p< 0.001), *‘congenital anomaly’* (ROR = 6.05, 95%CI: 6.03-6.07, p< 0.001), *‘disabling’* (ROR = 1.13, 95%CI: 1.10-1.15,p< 0.001) (not significant in males), *‘life threatening’* (ROR = 1.54, 95%CI: 1.52-1.56, p< 0.001) and *‘results in death’* (ROR = 1.34, 95%CI: 1.32-1.36, p< 0.001) seriousness criteria. On the other hand, they had a significant negative association with *‘other medically important condition’* seriousness criterion (ROR = 0.77, 95%CI: 0.76-0.78, p< 0.001).

New ASMs had a significant negative association with *‘congenital anomaly’* (ROR = 0.17, 95%CI: 0.14-0.19, p< 0.001) and *‘life threatening’* seriousness criteria (ROR = 0.65, 95%CI: 0.63-0.67, p< 0.001). They had a significant negative association in general (among females), but a significant positive association in males with *‘caused/prolonged hospitalisation’* (ROR = 0.76, 95%CI: 0.75-0.77, p< 0.001) and *‘results in death’* (ROR = 0.75, 95%CI: 0.73-0.76, p< 0.001). There was a significant positive association in general and among females with *‘other medically important condition’* (ROR = 1.30, 95%CI: 1.29-1.31, p< 0.001), however no significant association could be detected in males. *‘Disabling’* seriousness criterion had a significant negative association (ROR = 0.89, 95%CI: 0.87-0.91, p< 0.001) (in general and in males), though a significant positive association was found among females.

## Outcomes

The group aged 18–64 years had the highest number of PTs in all outcomes (Table 6).

**TABLE 6:** Number of reported sADRs in different outcomes criteria by age groups.

Age group	Outcome (number of sADRs)						
	Fatal	Not Recovered/Not Resolved	Recovered/Resolved	Recovered/Resolved with sequelae	Recovering/Resolving	Not specified/Unknown	All
0–2 years	1,287	2,640	7,265	305	2,327	14,161	27,985
3–17 years	2,499	8,894	24,307	483	10,671	47,840	94,694
18–64 years	25,310	79,945	124,428	2,766	50,185	268,095	550,729
65 years or older	8,030	30,034	46,688	1,093	19,498	91,738	197,081
Not Specified	4,565	16,251	17,481	316	5,473	136,513	180,599
All	41,619	137,764	220,169	4,963	88,154	55,8347	1,051,088

The most frequently reported outcome was *‘recovered/resolved’* (214,442 PTs; 20.4%) followed by the outcome of *‘not recovered/not resolved’* (135,970 PTs; 12.94%) and *‘recovering/resolving’* (85,623 PTs; 8.15%). The same trends were found in both males (88,494 PTs; 22.62%, 46,223 PTs; 11.82% and 36,927 PTs; 9.44%, respectively) and females (126,110 PTs; 20.88%, 87,689 PTs; 14.52% and 49,523 PTs; 8.20%, respectively). Of all the reported PTs, 3.89% had a *‘fatal’* outcome (4.835% and 3.36% in males and females respectively). All in all, 482,597 PTs had a reported outcome, of which 364,319 (75.49%) were reported by healthcare professionals.

In the subgroup **barbiturates** a significant positive association was noticed with *‘recovered/resolved’* and *‘recovering/resolving’*, and a parallel negative association with *‘not recovered/not resolved’ was found* (Table 7)*.* Besides this, positive association with *‘fatal’* (methylphenobarbital five-fold) outcome was shown.

**TABLE 7:** A and B Associations of outcome criteria by ASM chemical subgroups. A Outcome criterion with positive association of ASM chemical subgroups. B Outcome criterion with negative association of ASM chemical subgroups.

Criterion	ASM chemical subgroup	ROR	Lower 95%CI	Upper 95%CI	p-value	PRR	Lower 95%CI	Chi-square value
Not Recovered/Not Resolved	Benzodiazepines	1.23	1.21	1.25	<0.001	1.19	1.18	408.02
	Other Antiepileptics	1.50	1.49	1.52	<0.001	1.43	1.42	4,385.91
Recovered/Resolved	Barbiturates	1.35	1.32	1.38	<0.001	1.26	1.24	371.55
	Carboxamides	1.57	1.56	1.58	<0.001	1.41	1.40	4,214.37
	Fatty Acid derivatives	1.03	1.02	1.04	<0.001	1.02	1.01	20.84
	Hydantoins	1.38	1.36	1.41	<0.001	1.28	1.27	781.91
	Succinimides	1.32	1.21	1.42	<0.001	1.24	1.16	27.42
Recovered/Resolved with Sequelae	Carboxamides	1.20	1.12	1.28	<0.001	1.20	1.12	18.35
	Fatty Acid derivatives	1.11	1.04	1.19	<0.001	1.11	1.03	7.33
	Hydantoins	1.39	1.26	1.52	<0.001	1.39	1.26	26.02
	Succinimides	2.09	1.62	2.55	<0.001	2.08	1.61	10.04
Recovering/Resolving	Barbiturates	1.55	1.51	1.59	<0.001	1.49	1.45	416.16
	Carboxamides	2.17	2.15	2.18	<0.001	2.01	1.99	7,023.83
	Fatty Acid derivatives	1.25	1.23	1.27	<0.001	1.23	1.21	535.99
	Hydantoins	1.21	1.17	1.24	<0.001	1.19	1.16	108.67
	Succinimides	1.98	1.85	2.11	<0.001	1.85	1.73	110.13
Fatal	Barbiturates	2.02	1.97	2.07	<0.001	1.95	1.90	762.76
	Benzodiazepines	2.28	2.25	2.30	<0.001	2.18	2.15	3,591.52
	Hydantoins	1.55	1.51	1.59	<0.001	1.52	1.48	404.46
	Oxazolidines	4.66	4.07	5.25	<0.001	4.08	3.58	31.45

Criterion	ASM chemical subgroup	ROR	Lower 95%CI	Upper 95%CI	p-value	PRR	Lower 95%CI	Chi-square value
Not Recovered/Not Resolved	Barbiturates	0.55	0.50	0.60	<0.001	0.59	0.54	565.69
	Carboxamides	0.60	0.58	0.62	<0.001	0.64	0.62	2,231.61
	Fatty Acid derivatives	0.69	0.67	0.70	<0.001	0.72	0.70	1,713.20
	Hydantoins	0.65	0.61	0.68	<0.001	0.68	0.65	612.78
	Succinimides	0.85	0.70	0.99	<0.001	0.86	0.74	5.24
Recovered/Resolved	Other Antiepileptics	0.74	0.73	0.75	<0.001	0.79	0.78	3,726.41
Recovered/Resolved with Sequelae	Benzodiazepines	0.78	0.66	0.89	<0.001	0.78	0.66	18.21
	Other Antiepileptics	0.86	0.81	0.92	<0.001	0.86	0.81	25.20
Recovering/Resolving	Other Antiepileptics	0.77	0.76	0.79	<0.001	0.79	0.78	1,199.55
Fatal	Carboxamides	0.77	0.74	0.81	<0.001	0.78	0.74	220.41
	Fatty Acid derivatives	0.82	0.79	0.85	<0.001	0.82	0.80	172.88
	Other Antiepileptics	0.74	0.72	0.76	<0.001	0.75	0.73	864.57

Regarding **benzodiazepines**, there was a significant positive association with *‘fatal’* and *‘not recovered/not resolved’* outcome, while a significant negative association was found with outcomes of *‘recovered/resolved with sequelae’* (Table 7).

**Carboxamides** and **fatty acid derivatives** had the same pattern, there was a significant negative association with *‘fatal’* and *‘not recovered/not resolved’* outcomes, while a significant positive association was found with *‘recovered/resolved’*, *‘recovered/resolved with sequelae’* and *‘recovering/resolving’* showing a favourable outcome (Table 7).

Checking **hydantoins**, a significant positive association was recognized with *‘fatal’*, *‘recovered/resolved’*, *‘recovered/resolved with sequelae’* and *‘recovering/resolving’* outcomes. However, a significant negative association was recognized with *‘not recovered/not resolved’* outcomes (Table 7).

Regarding **other antiepileptics**, there was a significant positive association with *‘not recovered/not resolved’* outcomes, while a significant negative association was found with the other four outcomes (Table 7).

With respect to **oxazolidines**, *‘fatal’* outcomes had a significant positive association, which, among others, was the highest (trimethadione) (Table 7).

As for **succinimides**, a significant positive association was noticed with *‘recovered/resolved’*, *‘recovered/resolved with sequelae’* and *‘recovering/resolving’* outcomes. A significant negative association was found with *‘not recovered/not resolved’* outcome (Table 7).

It can be noticed that the most favourable negative association is linked to BRV and ESL. The worst outcome is seen with ethotion, trimethadione, methylphenobarbital, fosphenytion, CLZ and GBP (Supplementary Table S3).

*‘Fatal’* outcome of PTs was reported most frequently by the following ASMs: GBP (6,619; 16.21%), CLZ (5,994; 14.68%), PGB (5,671; 13.88%), VPA (4,221; 10.33%) and LEV (2,907; 7.12%). Among PTs with *‘not recovered/not resolved’* outcome, PGB (41,648; 30.63%), GBP (15,624; 11.49%), VPA (13,918; 10.24%), CLZ (11,186; 8.23%) and LEV (10,321; 7.59%) were the most common ones (Supplementary Table S3).

Old ASMs (Supplementary Table S4) had a significant negative association with outcome of *‘not recovered/not resolved’* (ROR = 0.71, 95%CI: 0.70-0.72, p< 0.001) showing the effectiveness of them. Noteworthy, there are differences between males and females. New ASMs had a significant positive association with ‘*not recovered/not resolved’* outcomes (ROR = 1.41, 95%CI: 1.40-1.42, p< 0.001). They had a significant negative association (same in females) and a significant positive association in males with *‘recovered/resolved’* (ROR = 0.75, 95%CI: 0.75-0.76, p< 0.001) and *‘recovered/resolved with sequelae’* (ROR = 0.85, 95%CI: 0.79-0.90, p< 0.001) outcomes. A significant negative association (ROR = 0.73, 95%CI: 0.71-0.75, p< 0.001) and a significant positive association in males were recognized with *‘fatal’* outcomes. Regarding ‘*recovering/resolving*’ outcomes, there was a significant negative association in general (ROR = 0.66, 95%CI: 0.65-0.68, p< 0.001), however no significant association was detected in males.

### Sudden unexpected death in epilepsy (SUDEP)

Out of all the reported PTs, 386 (0.04%) PTs were related to SUDEP, of which 358 (91.56%) were reported by healthcare professionals. As for males and females, 191 (0.05%) and 176 (0.03%) cases were reported as SUDEP, respectively. The number of reported SUDEP was as follows in the different age groups: 0–2 years: 11 (2.85%); 3–17 years: 50 (12.95%); 18–64 years: 249 (64.51%); 65 years or older: 2 (0.52%); not specified: 74 (19.17%). Age and gender were dependent variables χ^2^ (4, N = 276,694) = 97.6017, *p* < 0.0001 and χ^2^ (1, N = 276,694) = 15.9611, *p* = 0.000065, respectively.

There was a significant increased association with SUDEP (Table 8) as a reported PT in the subgroup of **barbiturates** (ROR = 1.84, 95%CI: 1.32-2.35, p< 0.001) (not significant in males) and **carboxamides** in males only (ROR = 1.58, 95%CI: 1.20-1.97, p< 0.001). As for **other antiepileptics**, there was no significant association in general, however males had a significant positive association (ROR = 1.56, 95%CI: 1.34-1.78, p< 0.001) and females had a significant negative association (ROR = 0.67, 95%CI: 0.45-0.89, p< 0.001).

**TABLE 8:** Sudden unexplained death in epilepsy **A** Sudden unexplained death by antiseizure medication chemical subgroups **B** Sudden unexplained death by antiseizure medications. **C** Sudden unexplained death by comparing old and new antiseizure medications.

ASM chemical subgroup	ROR	Lower 95%CI	Upper 95%CI	p-value	PRR	Lower 95%CI	Chi-square value
Barbiturates	1.84	1.32	2.35	<0.001	1.84	1.321706	5.51
Benzodiazepines	0.66	0.22	1.10	0.003	0.66	0.222455	3.43
Carboxamides	1.05	0.74	1.36	<0.001	1.05	0.740172	0.09
Fatty Acid derivatives	0.93	0.64	1.22	<0.001	0.93	0.644622	0.23
Hydantoins	1.29	0.83	1.75	<0.001	1.29	0.82858	1.18
Other Antiepileptics	1.00	0.80	1.21	<0.001	1.00	0.801031	0.00
Oxazolidines	0.00	N/C	N/C	N/C	0.00	N/C	0.03
Succinimides	1.42	N/C	3.39	N/C	1.42	N/C	0.13

ASM	ROR	Lower 95%CI	Upper 95%CI	p-value	PRR	Lower 95%CI	Chi-square value
Barbexaclone	40.31	38.91	41.71	<0.001	39.73	38.35	75.16
Brivaracetam	2.49	1.79	3.19	<0.001	2.48	1.78	6.95
Cenobamate	3.24	1.85	4.63	<0.001	3.24	1.85	3.08
Clobazam	2.02	1.40	2.65	<0.001	2.02	1.39	5.04
Eslicarbazepine	3.28	2.53	4.03	<0.001	3.28	2.53	10.87
Felbamate	8.01	6.62	9.40	<0.001	7.99	6.60	12.17
Fenfluramine	4.46	3.07	5.85	<0.001	4.46	3.07	5.34
Lacosamide	3.70	3.38	4.02	<0.001	3.70	3.38	75.30
Lamotrigine	1.88	1.60	2.16	<0.001	1.88	1.60	20.21
Levetiracetam	2.17	1.90	2.43	<0.001	2.16	1.90	33.96
Perampanel	5.85	5.30	6.41	<0.001	5.84	5.29	50.49
Phenobarbital	1.79	1.22	2.37	<0.001	1.79	1.22	4.06
Retigabine	15.96	15.26	16.67	<0.001	15.88	15.18	109.28
Stiripentol	2.91	1.52	4.30	<0.001	2.91	1.52	2.49
Sultiame	5.19	3.22	7.15	<0.001	5.18	3.22	3.37
Tiagabine	3.78	2.39	5.17	<0.001	3.77	2.39	4.06
Vigabatrin	3.62	3.12	4.12	<0.001	3.62	3.12	29.11

	ROR	Lower 95%CI	Upper 95%CI	z statistic	p-value	PRR	Chi-square value
Old ASMs	0.72	0.49	0.94	6.33	p< 0.001	0.72	8.78
*Male*	*0.51*	*0.15*	*0.87*	*2.81*	*p < 0.001*	*0.51*	*14.05*
*Female*	*0.78*	*0.49*	*1.06*	*5.38*	*p < 0.001*	*0.78*	*3.06*
New ASMs	1.40	1.17	1.62	12.34	p< 0.001	1.40	8.78
*Male*	*2.07*	*1.86*	*2.28*	*19.56*	*p < 0.001*	*2.07*	*49.38*
*Female*	*0.69*	*0.47*	*0.91*	*6.27*	*p < 0.001*	*0.69*	*11.51*

SUDEP as a PT was most commonly reported in association with the following ASMs: LEV (65; 16.84%), LTG (58; 15.03%), LCM (43; 11.14%), VPA (36; 9.33%) and CBZ (24; 6.22%) (Table 8).

Surprisingly, old type ASMs (Table 8) had a significant negative association (ROR = 0.72, 95%CI: 0.49-0.94, p< 0.001) (not significant in females), while new type ASMs had a significant positive association (ROR = 1.40, 95%CI: 1.17-1.62, p< 0.001) (same in males) and a significant negative association in females.

Detailed ASM can be found in Table 8B.

## Discussion

Considering the complexity of epilepsy treatment, together with many available ASMs to choose from, it became very challenging to choose the suitable ASM for each individual patient. Moreover, the ADRs caused by ASMs can be an important determining factor in ASM selection (Löscher and Klein, 2021). Due to the advancement in epilepsy treatment, there is a compelling need to study ADRs of ASMs either those which were recently introduced to the market or the traditional old ASMs, and to study their possible outcomes and seriousness.

Although EV is a very valuable source of sADRs, only few studies were conducted using it and they were also confined to a certain region or limited to a short time span. To our knowledge, the current study incorporated one of the longest time periods - 10 years - which resulted in a huge amount of data (276,694 reports including 1,051,142 sADRs reported in PTs), which provides evidential strength to the findings. Besides these, it covered the era of many new ASMs with sufficient time to have reports on their ADRs.

We could detect gender differences mainly on the reports of women. In a Norwegian population-based study of a previous time period found that two-thirds of all reports were from women, which is in line with our study (53.83% of reports came from females) (Baftiu et al., 2019).

In this study, most PTs were detected in the adult group aged between 18 and 64 years, which constituted 52.40% of all PTs and had the highest number of PTs in all outcomes and seriousness criteria. This might be because ASMs were prescribed for adults almost immediately after the medicines’ appearance on the market but were given to children only if they were safe. Moreover, ADR was reported as a consequence of adult ASM treatment, and it was identified as *‘congenital anomaly’* seriousness in the age group between 2 and 17 years, who had the highest percentage of PTs (27.05%). Pattern differences were also reported in a Norwegian study (Heger et al., 2022). The above was also emphasised in the publication by Landmark et al. who examined prescription patterns (Landmark et al., 2011). Nevertheless, we should remember that co-morbidity and polytherapy frequently occur among adults and may influence prescription, which, unfortunately, was not captured in this database.

From a chronological point of view, the highest number of PTs (150,689) was reported in 2021, followed by 2017, 2019 and 2020 in 120,695, 120,163 and 118,564 cases, respectively. Interestingly, the Annual Report on EV for the European Parliament stated that compared to earlier years, the number of reports rocketed in 2021 (Rothman et al., 2004). Based on the analysis, it was due to the authorized COVID-19 vaccines to support the enhanced monitoring pandemic plan. Nevertheless, data suggest the number of reported ADR increased in 2021, which was possibly associated not only with increased awareness of ADR reporting but also an increased number of potential interactions. The importance of the knowledge of interactions causing ADRs has been shown by the new online search engines for interaction in recent years (Liverpool Uo, 2024).

Concerning the group of all ASMs, the most frequently reported PTs were ‘*seizure’*, *‘drug ineffective’*, *‘somnolence’* and *‘dizziness’*. It is worth noting that withdrawal of ASMs/non-adherence to ASMs may cause seizure. Besides these, it is also important to mention that interaction decreases the effect of an ASM, may even cause ineffectiveness and/or seizure or may lead to intoxication also resulting in seizure, somnolence or dizziness. The high number of reported PTs suggests that patients had more likely no seizure control but seizure exacerbation, or paradoxical drug reaction taking an ASM (Gesche et al., 2021; Jaramillo et al., 2022). In the NorPD database, rash was the first most frequently reported PT, followed by dizziness, SUDEP, cross-sensitivity reaction and pyrexia (Baftiu et al., 2019).

The most frequently reported sADRs belonged to four SOCs: *‘nervous system disorders’*, *‘general disorders and administration site conditions’*, *‘psychiatric disorders’*. They showed a similar tendency in a previous publication whilst *‘injury, poisoning and procedural complications’* was 3.6-times higher (Baftiu et al., 2019). Nevertheless, the patterns of prescribed ASMs within the above mentioned SOC groups were different, where given. For example, a significant positive association with *‘nervous system disorders’* was found in fatty acid derivatives, hydantoins and succinimides pharmacological ASMs groups in our examination, but in a research by Baftiu, pregabalin was the most commonly mentioned ASM (Evans et al., 2001).

Although the SOC of *‘congenital, familial and genetic disorders’* constituted only 1.58% of all the reported PTs, it is useful to mention that significant positive associations were found in oxazolidines and fatty acid derivates; perhaps this finding in fatty acid derivatives can be justified by the fact of VPA belonging to this group with 91.08% of its reported PTs which is well known for its high teratogenicity risk (Tomson et al., 2019).

Two-thirds of PTs were reported on new ASMs (678,482 PTs; 64.55%), compared to old ASMs (372,660 PTs; 35.45%). This may be explained by raising awareness of the newly marketed ASMs and their stricter monitoring compared to that of the old ones as part of pharmacovigilance activity. This is why reports in the group of other ASMs, ‘*not recovered/not resolved*’ were overreported, as those reports came mostly from studies. In reported PTs, 75.49% of PTs had an outcome and 70.42% of PTs had seriousness criteria. As for seriousness, it is worth mentioning that 35.31% of PTs had seriousness of *‘caused/prolonged hospitalisation’* while 5.79% had seriousness criterion of *‘results in death’*. Old ASMs had a significant positive association with *‘caused/prolonged hospitalisation’*, *‘congenital anomaly’*, *‘disabling’*, *‘life threatening’* and *‘results in death’*. As for outcomes, only 20.4% of PTs had outcome of *‘recovered/resolved’* while 12.94% had the outcome of *‘not recovered/not resolved’* which ranked in the second place. Of all the reported PTs, 3.89% had *‘fatal’* outcome. We had more favourable outcome statistics compared to the study by Baftiu, which might contain overreported ADRs (Baftiu et al., 2019). Old ASMs had a significant negative association with outcome of *‘not recovered/not resolved’*, however an increased significant association was found with *‘fatal’* outcome. So, based on our analysis it can be concluded that more ADRs were reported for new ASMs, but more serious ADRs were captured for old ASMs. Concerning old ASMs, our study also showed an increase in significant associations with fatal outcomes and hospitalization, as well as congenital anomaly, disabling, life threatening and death seriousness criteria; still, a higher number of sADRs was found in new ASMs.

In terms of reported sADRs, female dominance was recorded, and regarding old vs. new ASMs, statistically significant correlation was detected. Nevertheless, many findings also showed similar associations among males.

SUDEP is a rare, but fatal event among PWEs, so even a smaller ratio of reported PTs - 386 (0.04%)—is considered as ‘high impact’. In a Norwegian study it was higher (2.13%), calling the attention to regional differences. Being the source of information, healthcare professionals reported 91.56% of all cases, which emphasises the above. Old ASMs had a significant negative association, while new ASMs had a significant positive association.

Nevertheless, it is an important finding that, regarding *‘fatal’* outcome and *‘results in death’* seriousness, our study found a significant positive association with old ASMs in general.

A few studies were conducted on the causality of specific ASMs regarding SUDEP (Tomson et al., 2005). These studies have highlighted the importance of adherence, polytherapy (three or more ASMs), besides many other non-pharmacological risk factors, e.g., type of the seizure (Sveinsson et al., 2020). Among the studies even fewer discussed the role of specific ASMs and, using ‘living controls’ (Nightscales et al., 2023), they found that LEV, LTG and VPA showed reduced risk for SUDEP. Other studies found LTG, VPA and CBZ listed among ASMs with the five highest numbers of SUDEP occurrence (Baftiu et al., 2019). These are contradictory findings. In contrast, RORs in our study were calculated and compared with all ASMs as control/background in the EV. At the moment we think more precise evaluation of specific ASMs are needed in the old and new summarized group.

A detailed summary from 2011 presented the comparative efficacy, safety, and tolerability of newer *versus* older ASMs (mainly CBZ or VPA and, to a lesser extent, PHT and sustained/controlled-release CBZ) (Talati et al., 2011). According to their findings of the summary from 2011, older ASMs such as VPA and CBZ had ADRs like somnolence, rash, fatigue more often than newer ASMs did. Also, newer ASMs did not significantly affect the risk of mortality *versus* their older counterparts, i.e., CBZ, PHT, or VPA.

According to data, PHT had a significant positive association, while CBZ had a significant negative association with SOC of *‘nervous system disorders’*.

## Main clinical implications

This study is based on the analysis of real-world data and may contribute to the selection of ASMs. The importance of international pharmacovigilance databases should be emphasized, as they could reveal rare but clinically relevant ADRs. Healthcare professionals are encouraged to report any ADRs in order to get a picture of the real situation. Clinical pharmacists can assist epileptologists in this regard. It is important to study the ADRs attributed to ASMs for healthcare professionals working with PWE because, especially the serious ones, could limit the use of ASMs. But it should be also noted that there is no medicine without ADR. The ADRs should be considered prior to prescribing an ASM, as they are one of the most important factors that influence tolerability when initiating treatment and may also lead to impaired adherence. Adherence is essential to achieve seizure control or seizure freedom, and as a life-threatening outcome status epilepticus may occur among non-adherent patients (Horváth et al., 2019). Similarly, our findings showed that improper ASM selection or inadequate dose resulted in ‘seizure’ and ‘drug ineffective’ PTs were more common. This may highlight the role of the clinical pharmacist in the team treating PWE, who may help the epileptologist tailor the treatment.

It should be kept in mind that new ASMs are not free form ADRs, but less serious and the outcome is favourable compared to old ASMs. SOC with their ROR by old and new ASMs may contribute in prescription decision making. The most common ADRs affect the CNS, and rash is among the top ten ADRs. Although rash is a sign of drug induced dermatological disease, some type of these diseases has a well-defined genetic background. In case of some ASMs, liver function should be monitored. In carboxamides, hepatobiliary disorders are due to carbamazepine. Fosphenytoin and phenobarbital are similar in terms of hepatotoxicity. Increased ROR of congenital anomaly linked to VPA, trimethadione, phenacemide and mephenytoin. Slightly increased risk was observed in case of clobazam, methylphenobarbital, phenobarbital, primidone and topiramate. These reports mainly linked to male offspring.

In our study, we reviewed a 10-year-period of reported sADRs of ASMs in the EV international database. The majority of PTs were serious in the EV. Old ASMs were generally more closely associated with undesired outcomes and seriousness among reported ICSR. Considering their expected seriousness and outcomes, the safety profiles of different ASMs can play a cardinal role in the selection of ASMs.

## Limitation

The authors are aware that the study has certain limitations. Epilepsy is not the exclusive diagnosis for prescribing ASMs. Mandatory reporting in EV is required only on serious sADRs. Many factors such as media coverage, public awareness, workload of healthcare professionals and others can influence reporting, which can lead to either under-reporting or over-reporting. Despite all that, the huge number of reports and studies on PTs, the majority of information was reported by healthcare professionals, which is also a strength of our study: in addition to the high number of reported PTs, it can support the evidence included in EV. It must be noted, that the number of patients taking ASM could not be given, so the prevalence of drug users and incidence of ADR cannot be counted. Nevertheless, we think these data also have importance, only we have to be aware of the limitations.

## Data Availability

The raw data supporting the conclusions of this article will be made available by the authors, without undue reservation.
