# Supplementary material for: Seriousness and outcomes of reported adverse drug reactions in old and new antiseizure medications: a pharmacovigilance study using EudraVigilance database
Source: Front Pharmacol. 2024 Jul 24;15:1411134. doi: 10.3389/fphar.2024.1411134 (PMC11307265; doi:10.3389/fphar.2024.1411134)
Supplement: Supplementary file 1 [file Table1.docx]

**Supplementary table 1 A and B** Associations of seriousness criteria by ASMs

**A** Seriousness criterion with positive association of ASMs

| **Criterion** | **ASM** | **ROR** | **Lower 95%CI** | **Upper 95%CI** | **p-value** | **PRR** | **Lower 95%CI** | **Chi-square value** |
| --- | --- | --- | --- | --- | --- | --- | --- | --- |
| **Caused/Prolonged Hospitalisation** | Carbamazepine | 1.83 | 1.82 | 1.85 | <0.001 | 2.04 | 2.03 | 5,172.57 |
|  | Clonazepam | 1.30 | 1.29 | 1.32 | <0.001 | 1.67 | 1.66 | 771.59 |
|  | Clorazepate potassium | 4.27 | 3.49 | 5.06 | <0.001 | 2.68 | 2.45 | 11.68 |
|  | Felbamate | 1.30 | 1.14 | 1.45 | <0.001 | 1.59 | 1.50 | 8.16 |
|  | Fenfluramine | 1.62 | 1.51 | 1.74 | <0.001 | 1.80 | 1.74 | 53.41 |
|  | Fosphenytoin | 1.36 | 1.27 | 1.45 | <0.001 | 1.63 | 1.58 | 32.29 |
|  | Lamotrigine | 1.73 | 1.72 | 1.75 | <0.001 | 1.99 | 1.98 | 4,321.50 |
|  | Mesuximide | 1.58 | 1.28 | 1.89 | <0.001 | 1.78 | 1.61 | 6.59 |
|  | Methylphenobarbital | 2.15 | 2.01 | 2.28 | <0.001 | 2.07 | 2.01 | 97.10 |
|  | Oxcarbazepine | 1.04 | 1.02 | 1.07 | <0.001 | 1.42 | 1.40 | 7.70 |
|  | Phenobarbital | 1.48 | 1.45 | 1.51 | <0.001 | 1.73 | 1.72 | 496.45 |
|  | Phenytoin | 1.60 | 1.58 | 1.62 | <0.001 | 1.83 | 1.82 | 1,466.58 |
|  | Primidone | 1.12 | 1.04 | 1.19 | <0.001 | 1.45 | 1.41 | 6.42 |
|  | Tiagabine | 2.51 | 2.40 | 2.61 | <0.001 | 2.22 | 2.17 | 233.30 |
|  | Vigabatrin | 1.49 | 1.45 | 1.52 | <0.001 | 1.73 | 1.71 | 347.11 |
|  | Zonisamide | 1.20 | 1.15 | 1.25 | <0.001 | 1.52 | 1.49 | 42.65 |
| **Congenital Anomaly** | Clobazam | 1.21 | 1.14 | 1.29 | <0.001 | 1.27 | 1.19 | 22.45 |
|  | Mephenytoin | 26.30 | 25.78 | 26.82 | <0.001 | 13.31 | 13.07 | 336.88 |
|  | Methylphenobarbital | 1.40 | 1.12 | 1.69 | <0.001 | 1.44 | 1.17 | 5.23 |
|  | Phenacemide | 26.71 | 25.62 | 27.80 | <0.001 | 13.41 | 12.90 | 76.73 |
|  | Phenobarbital | 1.72 | 1.67 | 1.78 | <0.001 | 1.77 | 1.72 | 328.83 |
|  | Primidone | 1.60 | 1.46 | 1.74 | <0.001 | 1.63 | 1.50 | 40.04 |
|  | Topiramate | 1.34 | 1.30 | 1.38 | <0.001 | 1.44 | 1.41 | 189.63 |
|  | Trimethadione | 34.37 | 33.89 | 34.85 | <0.001 | 14.95 | 14.76 | 521.92 |
|  | Valproic acid and Sodium Valproate | 12.96 | 12.94 | 12.98 | <0.001 | 12.47 | 12.45 | 80405.04 |
| **Disabling** | Clonazepam | 1.21 | 1.17 | 1.25 | <0.001 | 1.33 | 1.29 | 83.78 |
|  | Fenfluramine | 2.89 | 2.70 | 3.08 | <0.001 | 2.81 | 2.63 | 123.56 |
|  | Gabapentin | 1.15 | 1.12 | 1.19 | <0.001 | 1.30 | 1.27 | 58.26 |
|  | Pregabalin | 1.28 | 1.26 | 1.30 | <0.001 | 1.67 | 1.65 | 319.34 |
|  | Retigabine | 1.58 | 1.35 | 1.81 | <0.001 | 1.60 | 1.38 | 14.48 |
|  | Rufinamide | 1.36 | 1.06 | 1.66 | <0.001 | 1.39 | 1.10 | 3.85 |
|  | Valproic acid and Sodium Valproate | 1.62 | 1.59 | 1.65 | <0.001 | 1.89 | 1.86 | 1,111.04 |
| **Life Threatening** | Aminobutyric acid | 2.68 | 2.02 | 3.33 | <0.001 | 2.60 | 2.01 | 8.85 |
|  | Carbamazepine | 1.54 | 1.52 | 1.57 | <0.001 | 1.71 | 1.68 | 819.71 |
|  | Clonazepam | 1.82 | 1.79 | 1.84 | <0.001 | 1.95 | 1.93 | 1,468.43 |
|  | Clorazepate potassium | 17.79 | 17.08 | 18.51 | <0.001 | 10.42 | 10.04 | 113.01 |
|  | Fenfluramine | 1.44 | 1.21 | 1.67 | <0.001 | 1.48 | 1.27 | 9.52 |
|  | Fosphenytoin | 5.06 | 4.95 | 5.17 | <0.001 | 4.46 | 4.37 | 932.18 |
|  | Lamotrigine | 1.63 | 1.61 | 1.66 | <0.001 | 1.81 | 1.78 | 1,109.63 |
|  | Mephenytoin | 7.09 | 6.51 | 7.68 | <0.001 | 5.78 | 5.34 | 55.58 |
|  | Methylphenobarbital | 3.60 | 3.41 | 3.78 | <0.001 | 3.36 | 3.20 | 197.30 |
|  | Oxcarbazepine | 1.13 | 1.07 | 1.18 | <0.001 | 1.20 | 1.15 | 16.35 |
|  | Phenobarbital | 1.60 | 1.54 | 1.65 | <0.001 | 1.65 | 1.60 | 248.84 |
|  | Phenytoin | 1.38 | 1.34 | 1.43 | <0.001 | 1.47 | 1.44 | 210.99 |
|  | Tiagabine | 1.85 | 1.66 | 2.04 | <0.001 | 1.86 | 1.69 | 40.36 |
|  | Valproic acid and Sodium valproate | 1.04 | 1.02 | 1.07 | <0.001 | 1.25 | 1.23 | 8.99 |
|  | Zonisamide | 1.59 | 1.50 | 1.68 | <0.001 | 1.63 | 1.55 | 101.99 |
| **Other Medically Important Condition** | Aminobutyric acid | 2.89 | 2.35 | 3.44 | <0.001 | 2.17 | 2.07 | 10.07 |
|  | Barbexaclone | 3.43 | 2.97 | 3.88 | <0.001 | 2.23 | 2.16 | 19.79 |
|  | Ethotoin | 2.12 | 1.41 | 2.83 | <0.001 | 2.03 | 1.86 | 2.80 |
|  | Felbamate | 1.28 | 1.12 | 1.44 | <0.001 | 1.76 | 1.70 | 5.90 |
|  | Fosphenytoin | 1.15 | 1.05 | 1.24 | <0.001 | 1.69 | 1.66 | 5.23 |
|  | Gabapentin | 1.41 | 1.39 | 1.42 | <0.001 | 1.92 | 1.91 | 1,323.27 |
|  | Oxcarbazepine | 1.17 | 1.14 | 1.20 | <0.001 | 1.73 | 1.72 | 86.63 |
|  | Phenytoin | 1.36 | 1.34 | 1.38 | <0.001 | 1.83 | 1.82 | 467.68 |
|  | Pregabalin | 1.44 | 1.43 | 1.45 | <0.001 | 2.14 | 2.14 | 2,908.30 |
|  | Retigabine | 1.35 | 1.24 | 1.46 | <0.001 | 1.79 | 1.75 | 17.31 |
|  | Topiramate | 1.24 | 1.22 | 1.26 | <0.001 | 1.79 | 1.78 | 292.72 |
| **Results in Death** | Clonazepam | 2.24 | 2.21 | 2.26 | <0.001 | 2.36 | 2.34 | 3,721.90 |
|  | Clorazepate potassium | 10.84 | 10.11 | 11.57 | <0.001 | 7.31 | 6.87 | 60.80 |
|  | Ethotoin | 3.83 | 3.06 | 4.60 | <0.001 | 3.48 | 2.86 | 12.78 |
|  | Fenfluramine | 1.26 | 1.04 | 1.48 | <0.001 | 1.31 | 1.11 | 4.06 |
|  | Fosphenytoin | 3.02 | 2.90 | 3.15 | <0.001 | 2.87 | 2.76 | 318.25 |
|  | Gabapentin | 1.66 | 1.64 | 1.68 | <0.001 | 1.85 | 1.83 | 1,496.41 |
|  | Lamotrigine | 1.05 | 1.02 | 1.08 | <0.001 | 1.20 | 1.18 | 9.46 |
|  | Methylphenobarbital | 5.42 | 5.27 | 5.57 | <0.001 | 4.57 | 4.45 | 550.11 |
|  | Phenobarbital | 2.13 | 2.08 | 2.18 | <0.001 | 2.15 | 2.11 | 994.52 |
|  | Phenytoin | 1.67 | 1.64 | 1.71 | <0.001 | 1.76 | 1.73 | 738.64 |
|  | Primidone | 1.18 | 1.04 | 1.32 | <0.001 | 1.24 | 1.10 | 4.86 |
|  | Trimethadione | 6.51 | 5.99 | 7.03 | <0.001 | 5.22 | 4.85 | 62.91 |
|  | Vigabatrin | 1.56 | 1.50 | 1.62 | <0.001 | 1.62 | 1.56 | 179.06 |
|  | Zonisamide | 1.10 | 1.00 | 1.19 | <0.001 | 1.16 | 1.07 | 3.57 |

**B** Seriousness criterion with negative association of ASMs

| **Condition** | **ASM** | **ROR** | **Lower 95%CI** | **Upper 95%CI** | **p-value** | **PRR** | **Lower 95%CI** | **Upper 95%CI** | **Chi-square value** |
| --- | --- | --- | --- | --- | --- | --- | --- | --- | --- |
| Caused/Prolonged Hospitalisation | Brivaracetam | 0.32 | 0.26 | 0.38 | <0.001 | 0.57 | 0.52 | 0.62 | 1,179.66 |
|  | Cannabidiol | 0.52 | 0.48 | 0.56 | <0.001 | 0.86 | 0.83 | 0.89 | 696.84 |
|  | Cenobamate | 0.76 | 0.65 | 0.87 | <0.001 | 1.13 | 1.05 | 1.20 | 19.28 |
|  | Clobazam | 0.94 | 0.91 | 0.98 | <0.001 | 1.32 | 1.29 | 1.34 | 7.23 |
|  | Eslicarbazepine | 0.59 | 0.53 | 0.65 | <0.001 | 0.94 | 0.89 | 0.98 | 225.50 |
|  | Ethosuximide | 0.74 | 0.64 | 0.85 | <0.001 | 1.11 | 1.03 | 1.18 | 23.36 |
|  | Gabapentin | 0.92 | 0.90 | 0.93 | <0.001 | 1.37 | 1.36 | 1.38 | 103.58 |
|  | Lacosamide | 0.86 | 0.83 | 0.88 | <0.001 | 1.25 | 1.24 | 1.27 | 125.48 |
|  | Levetiracetam | 0.87 | 0.85 | 0.88 | <0.001 | 1.32 | 1.31 | 1.33 | 245.12 |
|  | Perampanel | 0.90 | 0.85 | 0.95 | <0.001 | 1.27 | 1.23 | 1.30 | 11.13 |
|  | Pregabalin | 0.66 | 0.65 | 0.67 | <0.001 | 1.25 | 1.25 | 1.26 | 3,988.17 |
|  | Retigabine | 0.81 | 0.70 | 0.93 | <0.001 | 1.18 | 1.10 | 1.26 | 9.20 |
|  | Stiripentol | 0.74 | 0.64 | 0.85 | <0.001 | 1.11 | 1.04 | 1.18 | 24.58 |
|  | Topiramate | 0.72 | 0.70 | 0.74 | <0.001 | 1.13 | 1.11 | 1.14 | 715.84 |
|  | Valproic acid and Sodium Valproate | 0.84 | 0.83 | 0.85 | <0.001 | 1.35 | 1.34 | 1.36 | 524.06 |
| Congenital Anomaly | Carbamazepine | 0.67 | 0.63 | 0.71 | <0.001 | 0.77 | 0.73 | 0.81 | 332.00 |
|  | Clonazepam | 0.29 | 0.22 | 0.36 | <0.001 | 0.33 | 0.27 | 0.40 | 1,370.33 |
|  | Gabapentin | 0.17 | 0.09 | 0.24 | <0.001 | 0.20 | 0.13 | 0.27 | 2,612.47 |
|  | Lacosamide | 0.20 | 0.09 | 0.31 | <0.001 | 0.22 | 0.11 | 0.34 | 881.93 |
|  | Lamotrigine | 0.80 | 0.77 | 0.84 | <0.001 | 0.92 | 0.89 | 0.96 | 115.67 |
|  | Levetiracetam | 0.56 | 0.52 | 0.61 | <0.001 | 0.65 | 0.61 | 0.69 | 608.78 |
|  | Oxcarbazepine | 0.40 | 0.31 | 0.50 | <0.001 | 0.44 | 0.35 | 0.54 | 351.28 |
|  | Phenytoin | 0.46 | 0.39 | 0.53 | <0.001 | 0.51 | 0.44 | 0.58 | 431.08 |
|  | Zonisamide | 0.65 | 0.51 | 0.79 | <0.001 | 0.69 | 0.56 | 0.83 | 35.68 |
| Disabling | Brivaracetam | 0.35 | 0.17 | 0.54 | <0.001 | 0.38 | 0.19 | 0.56 | 124.80 |
|  | Cannabidiol | 0.32 | 0.15 | 0.48 | <0.001 | 0.34 | 0.18 | 0.50 | 201.78 |
|  | Carbamazepine | 0.57 | 0.52 | 0.62 | <0.001 | 0.65 | 0.60 | 0.70 | 494.57 |
|  | Clobazam | 0.66 | 0.54 | 0.77 | <0.001 | 0.70 | 0.59 | 0.81 | 52.24 |
|  | Eslicarbazepine | 0.36 | 0.13 | 0.59 | 0.002 | 0.38 | 0.16 | 0.61 | 82.02 |
|  | Lacosamide | 0.59 | 0.51 | 0.66 | <0.001 | 0.64 | 0.56 | 0.71 | 188.87 |
|  | Levetiracetam | 0.58 | 0.53 | 0.62 | <0.001 | 0.66 | 0.61 | 0.71 | 483.96 |
|  | Oxcarbazepine | 0.58 | 0.50 | 0.67 | <0.001 | 0.63 | 0.54 | 0.71 | 140.96 |
|  | Perampanel | 0.67 | 0.51 | 0.84 | <0.001 | 0.71 | 0.55 | 0.87 | 22.08 |
|  | Phenobarbital | 0.69 | 0.60 | 0.79 | <0.001 | 0.74 | 0.65 | 0.83 | 56.11 |
|  | Primidone | 0.78 | 0.57 | 1.00 | <0.001 | 0.82 | 0.61 | 1.03 | 4.81 |
|  | Stiripentol | 0.39 | 0.00 | 0.78 | 0.048 | 0.41 | 0.03 | 0.79 | 23.63 |
|  | Vigabatrin | 0.26 | 0.07 | 0.44 | 0.006 | 0.28 | 0.10 | 0.46 | 231.24 |
| Life Threatening | Brivaracetam | 0.34 | 0.18 | 0.50 | <0.001 | 0.37 | 0.21 | 0.53 | 172.46 |
|  | Cannabidiol | 0.18 | 0.00 | 0.37 | 0.048 | 0.20 | 0.02 | 0.39 | 385.96 |
|  | Cenobamate | 0.44 | 0.10 | 0.77 | 0.01 | 0.47 | 0.14 | 0.80 | 23.88 |
|  | Clobazam | 0.79 | 0.70 | 0.88 | <0.001 | 0.85 | 0.76 | 0.93 | 24.55 |
|  | Eslicarbazepine | 0.72 | 0.58 | 0.86 | <0.001 | 0.77 | 0.63 | 0.90 | 20.00 |
|  | Gabapentin | 0.79 | 0.76 | 0.83 | <0.001 | 0.92 | 0.88 | 0.95 | 151.68 |
|  | Lacosamide | 0.68 | 0.62 | 0.74 | <0.001 | 0.75 | 0.69 | 0.81 | 142.08 |
|  | Levetiracetam | 0.84 | 0.80 | 0.87 | <0.001 | 0.96 | 0.93 | 1.00 | 85.65 |
|  | Pregabalin | 0.52 | 0.49 | 0.54 | <0.001 | 0.71 | 0.68 | 0.73 | 1,855.25 |
|  | Primidone | 0.54 | 0.31 | 0.76 | <0.001 | 0.58 | 0.36 | 0.79 | 29.26 |
|  | Stiripentol | 0.60 | 0.32 | 0.87 | <0.001 | 0.64 | 0.37 | 0.90 | 13.36 |
|  | Topiramate | 0.83 | 0.78 | 0.87 | <0.001 | 0.91 | 0.87 | 0.96 | 57.63 |
|  | Vigabatrin | 0.36 | 0.22 | 0.49 | <0.001 | 0.39 | 0.26 | 0.52 | 226.41 |
| Other Medically Important Condition | Brivaracetam | 0.35 | 0.30 | 0.39 | <0.001 | 0.92 | 0.89 | 0.95 | 1,510.47 |
|  | Cannabidiol | 0.25 | 0.21 | 0.29 | <0.001 | 0.74 | 0.72 | 0.77 | 3,447.38 |
|  | Carbamazepine | 0.77 | 0.75 | 0.78 | <0.001 | 1.52 | 1.51 | 1.52 | 806.55 |
|  | Cenobamate | 0.28 | 0.17 | 0.38 | <0.001 | 0.79 | 0.72 | 0.87 | 402.45 |
|  | Ethosuximide | 0.74 | 0.64 | 0.83 | <0.001 | 1.41 | 1.36 | 1.45 | 24.67 |
|  | Fenfluramine | 0.67 | 0.56 | 0.79 | <0.001 | 1.34 | 1.29 | 1.40 | 29.92 |
|  | Lacosamide | 0.71 | 0.69 | 0.73 | <0.001 | 1.41 | 1.40 | 1.42 | 605.38 |
|  | Lamotrigine | 0.94 | 0.92 | 0.95 | <0.001 | 1.65 | 1.65 | 1.66 | 51.80 |
|  | Levetiracetam | 0.98 | 0.96 | 0.99 | <0.001 | 1.68 | 1.67 | 1.68 | 6.13 |
|  | Methylphenobarbital | 0.72 | 0.59 | 0.86 | <0.001 | 1.39 | 1.33 | 1.45 | 14.43 |
|  | Perampanel | 0.34 | 0.29 | 0.40 | <0.001 | 0.91 | 0.88 | 0.95 | 1,078.05 |
|  | Phenobarbital | 0.85 | 0.82 | 0.88 | <0.001 | 1.51 | 1.50 | 1.53 | 71.76 |
|  | Primidone | 0.68 | 0.61 | 0.75 | <0.001 | 1.35 | 1.31 | 1.38 | 73.52 |
|  | Stiripentol | 0.28 | 0.18 | 0.38 | <0.001 | 0.80 | 0.73 | 0.87 | 441.27 |
|  | Sultiame | 0.43 | 0.26 | 0.61 | <0.001 | 1.05 | 0.95 | 1.16 | 57.36 |
|  | Tiagabine | 0.69 | 0.59 | 0.80 | <0.001 | 1.36 | 1.31 | 1.41 | 30.20 |
|  | Trimethadione | 0.53 | 0.05 | 1.00 | 0.028 | 1.18 | 0.92 | 1.44 | 4.61 |
|  | Valproic acid and Sodium Valproate | 0.78 | 0.77 | 0.80 | <0.001 | 1.59 | 1.59 | 1.60 | 1,001.82 |
|  | Vigabatrin | 0.87 | 0.84 | 0.91 | <0.001 | 1.53 | 1.51 | 1.54 | 34.25 |
|  | Zonisamide | 0.84 | 0.80 | 0.89 | <0.001 | 1.50 | 1.48 | 1.52 | 33.40 |
| Results in Death | Brivaracetam | 0.26 | 0.09 | 0.43 | 0.002 | 0.29 | 0.13 | 0.46 | 268.70 |
|  | Cannabidiol | 0.74 | 0.66 | 0.83 | <0.001 | 0.81 | 0.73 | 0.89 | 43.15 |
|  | Carbamazepine | 0.89 | 0.86 | 0.92 | <0.001 | 1.03 | 1.00 | 1.06 | 49.30 |
|  | Clobazam | 0.75 | 0.67 | 0.84 | <0.001 | 0.82 | 0.74 | 0.90 | 42.21 |
|  | Eslicarbazepine | 0.25 | 0.03 | 0.46 | 0.02 | 0.27 | 0.06 | 0.48 | 186.14 |
|  | Felbamate | 0.57 | 0.16 | 0.99 | 0.007 | 0.62 | 0.22 | 1.02 | 6.69 |
|  | Lacosamide | 0.91 | 0.87 | 0.96 | <0.001 | 1.00 | 0.96 | 1.05 | 12.07 |
|  | Levetiracetam | 0.96 | 0.93 | 0.99 | <0.001 | 1.10 | 1.08 | 1.13 | 6.90 |
|  | Oxcarbazepine | 0.82 | 0.76 | 0.87 | <0.001 | 0.89 | 0.84 | 0.95 | 43.31 |
|  | Perampanel | 0.37 | 0.20 | 0.54 | <0.001 | 0.41 | 0.24 | 0.57 | 135.38 |
|  | Pregabalin | 0.54 | 0.52 | 0.57 | <0.001 | 0.76 | 0.73 | 0.78 | 1,976.62 |
|  | Retigabine | 0.47 | 0.15 | 0.79 | 0.004 | 0.52 | 0.21 | 0.83 | 21.09 |
|  | Stiripentol | 0.52 | 0.26 | 0.79 | <0.001 | 0.57 | 0.32 | 0.82 | 22.93 |
|  | Tiagabine | 0.48 | 0.17 | 0.79 | 0.003 | 0.52 | 0.22 | 0.83 | 21.21 |
|  | Topiramate | 0.76 | 0.72 | 0.80 | <0.001 | 0.85 | 0.81 | 0.89 | 137.74 |
|  | Valproic acid and Sodium Valproate | 0.77 | 0.74 | 0.79 | <0.001 | 0.94 | 0.92 | 0.97 | 325.25 |

ASM: antiseizure medication, ROR: Reporting Odds Ratio, PRR: Proportional Reporting Ratio
