# Supplementary material for: Seriousness and outcomes of reported adverse drug reactions in old and new antiseizure medications: a pharmacovigilance study using EudraVigilance database
Source: Front Pharmacol. 2024 Jul 24;15:1411134. doi: 10.3389/fphar.2024.1411134 (PMC11307265; doi:10.3389/fphar.2024.1411134)
Supplement: Supplementary file 2 [file Table2.docx]

**Supplementary table 2** Comparison old and new ASMs by seriousness of PTs

| **Seriousness criterion** |  | **ROR** | **Lower 95%CI** | **Upper 95%CI** | **z statistic** | **p-value** | **PRR** | **Chi-square value** |
| --- | --- | --- | --- | --- | --- | --- | --- | --- |
| **Other Medically Important Condition** | Old ASMs | 0.77 | 0.76 | 0.78 | 185.50 | <0.001 | 0.90 | 4,027.59 |
|  | *Male* | *0.77* | *0.75* | *0.78* | *140.72* | *<0.001* | *0.89* | *2,420.14* |
|  | *Female* | *0.86* | *0.85* | *0.87* | *166.05* | *<0.001* | *0.94* | *921.86* |
|  | New ASMs | 1.30 | 1.29 | 1.31 | 313.77 | <0.001 | 1.11 | 4,027.59 |
|  | *Male* | *0.99* | *0.98* | *1.00* | *205.92* | *<0.001* | *1.00* | *1.61* |
|  | *Female* | *1.27* | *1.26* | *1.27* | *308.38* | *<0.001* | *1.10* | *3,304.22* |
| **Caused/Prolonged Hospitalisation** | Old ASMs | 1.32 | 1.31 | 1.32 | 310.84 | <0.001 | 1.19 | 4,200.00 |
|  | *Male* | *1.28* | *1.27* | *1.29* | *231.20* | *<0.001* | *1.17* | *1,987.03* |
|  | *Female* | *1.37* | *1.36* | *1.38* | *264.09* | *<0.001* | *1.22* | *3,739.14* |
|  | New ASMs | 0.76 | 0.75 | 0.77 | 179.74 | <0.001 | 0.84 | 4,200.00 |
|  | *Male* | *1.07* | *1.06* | *1.08* | *218.25* | *<0.001* | *1.05* | *206.84* |
|  | *Female* | *0.81* | *0.80* | *0.82* | *191.96* | *<0.001* | *0.87* | *2,565.15* |
| **Congenital Anomaly** | Old ASMs | 6.05 | 6.03 | 6.07 | 536.30 | <0.001 | 5.60 | 32,116.80 |
|  | *Male* | *3.05* | *3.03* | *3.07* | *291.97* | *<0.001* | *2.86* | *12,476.78* |
|  | *Female* | *1.27* | *1.24* | *1.29* | *106.34* | *<0.001* | *1.25* | *392.83* |
|  | New ASMs | 0.17 | 0.14 | 0.19 | 14.66 | <0.001 | 0.18 | 32,116.80 |
|  | *Male* | *0.40* | *0.37* | *0.43* | *25.26* | *<0.001* | *0.41* | *3,547.28* |
|  | *Female* | *0.10* | *0.06* | *0.14* | *5.06* | *<0.001* | *0.11* | *21,053.38* |
| **Disabling** | Old ASMs | 1.13 | 1.10 | 1.15 | 103.16 | <0.001 | 1.12 | 118.26 |
|  | *Male* | *1.01* | *0.98* | *1.04* | *69.32* | *<0.001* | *1.01* | *0.70* |
|  | *Female* | *1.09* | *1.06* | *1.11* | *80.73* | *<0.001* | *1.08* | *39.13* |
|  | New ASMs | 0.89 | 0.87 | 0.91 | 81.37 | <0.001 | 0.89 | 118.26 |
|  | *Male* | *0.94* | *0.91* | *0.96* | *71.80* | *<0.001* | *0.94* | *24.25* |
|  | *Female* | *1.03* | *1.00* | *1.05* | *94.89* | *<0.001* | *1.02* | *5.53* |
| **Life Threatening** | Old ASMs | 1.54 | 1.52 | 1.56 | 165.77 | <0.001 | 1.51 | 2,188.48 |
|  | *Male* | *1.29* | *1.26* | *1.31* | *108.67* | *<0.001* | *1.27* | *457.57* |
|  | *Female* | *1.58* | *1.56* | *1.61* | *148.13* | *<0.001* | *1.55* | *1,881.26* |
|  | New ASMs | 0.65 | 0.63 | 0.67 | 69.92 | <0.001 | 0.66 | 2,188.48 |
|  | *Male* | *0.95* | *0.92* | *0.97* | *83.42* | *<0.001* | *0.95* | *23.78* |
|  | *Female* | *0.73* | *0.71* | *0.74* | *73.77* | *<0.001* | *0.74* | *1,074.02* |
| **Results in Death** | Old ASMs | 1.34 | 1.32 | 1.36 | 158.10 | <0.001 | 1.32 | 1,200.79 |
|  | *Male* | *1.39* | *1.37* | *1.41* | *132.45* | *<0.001* | *1.36* | *1,002.95* |
|  | *Female* | *1.12* | *1.10* | *1.15* | *106.72* | *<0.001* | *1.12* | *124.53* |
|  | New ASMs | 0.75 | 0.73 | 0.76 | 88.02 | <0.001 | 0.76 | 1,200.79 |
|  | *Male* | *1.30* | *1.28* | *1.32* | *136.64* | *<0.001* | *1.28* | *769.98* |
|  | *Female* | *0.60* | *0.58* | *0.62* | *65.29* | *<0.001* | *0.62* | *3,153.48* |

ASM: antiseizure medication, PT: preferred term, ROR: Reporting Odds Ratio, PRR: Proportional Reporting Ratio
