# Supplementary material for: Seriousness and outcomes of reported adverse drug reactions in old and new antiseizure medications: a pharmacovigilance study using EudraVigilance database
Source: Front Pharmacol. 2024 Jul 24;15:1411134. doi: 10.3389/fphar.2024.1411134 (PMC11307265; doi:10.3389/fphar.2024.1411134)
Supplement: Supplementary file 3 [file Table3.docx]

**Supplementary table 3 A and B** Associations of outcome criteria by ASMs

**A** Outcome criterion with positive association of ASMs

| **Criterion** | **ASM** | **ROR** | **Lower 95%CI** | **Upper 95%CI** | **p-value** | **PRR** | **Lower 95%CI** | **Chi-square value** |
| --- | --- | --- | --- | --- | --- | --- | --- | --- |
| **Not Recovered/Not Resolved** | Brivaracetam | 1.54 | 1.48 | 1.59 | <0.001 | 1.44 | 1.39 | 246.64 |
|  | Cannabidiol | 1.37 | 1.32 | 1.41 | <0.001 | 1.31 | 1.27 | 171.77 |
|  | Cenobamate | 3.15 | 3.04 | 3.25 | <0.001 | 2.46 | 2.39 | 527.23 |
|  | Clonazepam | 1.32 | 1.30 | 1.34 | <0.001 | 1.27 | 1.25 | 659.73 |
|  | Eslicarbazepine | 1.13 | 1.05 | 1.20 | <0.001 | 1.11 | 1.05 | 10.17 |
|  | Fenfluramine | 1.54 | 1.39 | 1.68 | <0.001 | 1.44 | 1.32 | 34.30 |
|  | Gabapentin | 1.38 | 1.36 | 1.40 | <0.001 | 1.32 | 1.30 | 1,200.75 |
|  | Lacosamide | 1.26 | 1.23 | 1.29 | <0.001 | 1.22 | 1.19 | 227.27 |
|  | Pheneturide | 6.73 | 5.34 | 8.12 | <0.001 | 3.87 | 3.17 | 9.76 |
|  | Pregabalin | 1.66 | 1.65 | 1.67 | <0.001 | 1.54 | 1.53 | 6,323.04 |
| **Recovered/Resolved** | Aminobutyric acid | 1.79 | 1.33 | 2.24 | <0.001 | 1.54 | 1.23 | 6.40 |
|  | Cannabidiol | 1.21 | 1.17 | 1.26 | <0.001 | 1.16 | 1.13 | 85.20 |
|  | Carbamazepine | 1.62 | 1.61 | 1.64 | <0.001 | 1.45 | 1.44 | 3,793.38 |
|  | Cenobamate | 1.37 | 1.26 | 1.48 | <0.001 | 1.28 | 1.20 | 32.64 |
|  | Clobazam | 1.08 | 1.04 | 1.12 | <0.001 | 1.06 | 1.03 | 12.58 |
|  | Clorazepate potassium | 2.26 | 1.52 | 3.00 | <0.001 | 1.80 | 1.33 | 4.89 |
|  | Eslicarbazepine | 1.20 | 1.14 | 1.26 | <0.001 | 1.15 | 1.11 | 35.29 |
|  | Ethosuximide | 1.32 | 1.21 | 1.43 | <0.001 | 1.24 | 1.16 | 25.18 |
|  | Fosphenytoin | 1.96 | 1.87 | 2.06 | <0.001 | 1.64 | 1.58 | 199.88 |
|  | Levetiracetam | 1.05 | 1.03 | 1.07 | <0.001 | 1.04 | 1.03 | 33.26 |
|  | Mesuximide | 1.36 | 1.02 | 1.71 | <0.001 | 1.27 | 1.01 | 3.10 |
|  | Methylphenobarbital | 1.70 | 1.56 | 1.85 | <0.001 | 1.49 | 1.39 | 53.04 |
|  | Oxcarbazepine | 1.33 | 1.30 | 1.36 | <0.001 | 1.24 | 1.22 | 370.47 |
|  | Perampanel | 2.31 | 2.25 | 2.36 | <0.001 | 1.82 | 1.79 | 1,049.20 |
|  | Pheneturide | 3.90 | 2.52 | 5.29 | <0.001 | 2.45 | 1.76 | 4.32 |
|  | Phenobarbital | 1.33 | 1.30 | 1.36 | <0.001 | 1.25 | 1.22 | 278.83 |
|  | Phenytoin | 1.36 | 1.33 | 1.38 | <0.001 | 1.27 | 1.25 | 647.67 |
|  | Primidone | 1.36 | 1.27 | 1.44 | <0.001 | 1.26 | 1.20 | 54.36 |
|  | Retigabine | 1.65 | 1.54 | 1.77 | <0.001 | 1.46 | 1.38 | 74.61 |
|  | Rufinamide | 1.64 | 1.50 | 1.78 | <0.001 | 1.45 | 1.35 | 48.47 |
|  | Stiripentol | 1.32 | 1.21 | 1.42 | <0.001 | 1.24 | 1.16 | 27.00 |
|  | Sultiame | 1.81 | 1.63 | 2.00 | <0.001 | 1.56 | 1.43 | 41.13 |
|  | Tiagabine | 3.77 | 3.67 | 3.88 | <0.001 | 2.41 | 2.36 | 725.83 |
|  | Topiramate | 1.09 | 1.06 | 1.11 | <0.001 | 1.07 | 1.05 | 52.13 |
|  | Valproic acid and Sodium Valproate | 1.05 | 1.03 | 1.06 | <0.001 | 1.04 | 1.03 | 43.65 |
|  | Zonisamide | 1.47 | 1.42 | 1.52 | <0.001 | 1.34 | 1.30 | 218.43 |
| **Recovered/Resolved with Sequelae** | Aminobutyric acid | 7.82 | 6.67 | 8.98 | <0.001 | 7.59 | 6.47 | 17.22 |
|  | Carbamazepine | 1.34 | 1.25 | 1.44 | <0.001 | 1.34 | 1.25 | 40.77 |
|  | Ethosuximide | 2.30 | 1.83 | 2.76 | <0.001 | 2.28 | 1.82 | 13.00 |
|  | Fenfluramine | 4.20 | 3.79 | 4.62 | <0.001 | 4.14 | 3.74 | 54.83 |
|  | Lamotrigine | 1.11 | 1.01 | 1.20 | <0.001 | 1.10 | 1.01 | 4.09 |
|  | Perampanel | 1.49 | 1.19 | 1.80 | <0.001 | 1.49 | 1.19 | 6.71 |
|  | Phenytoin | 1.39 | 1.26 | 1.52 | <0.001 | 1.38 | 1.25 | 24.35 |
|  | Primidone | 1.57 | 1.15 | 1.99 | <0.001 | 1.56 | 1.14 | 4.44 |
|  | Topiramate | 1.14 | 1.01 | 1.27 | <0.001 | 1.14 | 1.01 | 4.08 |
|  | Valproic acid and Sodium Valproate | 1.15 | 1.07 | 1.23 | <0.001 | 1.15 | 1.07 | 12.59 |
| **Recovering/Resolving** | Carbamazepine | 2.35 | 2.33 | 2.37 | <0.001 | 2.15 | 2.13 | 7,193.68 |
|  | Cenobamate | 1.57 | 1.42 | 1.72 | <0.001 | 1.50 | 1.37 | 34.33 |
|  | Clorazepate potassium | 2.45 | 1.49 | 3.41 | <0.001 | 2.21 | 1.41 | 3.57 |
|  | Ethosuximide | 2.10 | 1.97 | 2.23 | <0.001 | 1.94 | 1.82 | 123.14 |
|  | Ethotoin | 9.19 | 8.58 | 9.80 | <0.001 | 5.68 | 5.33 | 75.02 |
|  | Fosphenytoin | 1.56 | 1.42 | 1.71 | <0.001 | 1.50 | 1.37 | 38.59 |
|  | Lamotrigine | 1.16 | 1.14 | 1.19 | <0.001 | 1.15 | 1.13 | 141.97 |
|  | Oxcarbazepine | 1.51 | 1.47 | 1.55 | <0.001 | 1.45 | 1.42 | 398.64 |
|  | Perampanel | 1.92 | 1.85 | 2.00 | <0.001 | 1.80 | 1.73 | 315.32 |
|  | Phenobarbital | 1.59 | 1.54 | 1.63 | <0.001 | 1.52 | 1.48 | 389.37 |
|  | Phenytoin | 1.18 | 1.15 | 1.22 | <0.001 | 1.17 | 1.13 | 81.81 |
|  | Primidone | 1.45 | 1.34 | 1.57 | <0.001 | 1.41 | 1.30 | 40.92 |
|  | Retigabine | 1.78 | 1.62 | 1.94 | <0.001 | 1.68 | 1.54 | 51.99 |
|  | Rufinamide | 2.56 | 2.39 | 2.73 | <0.001 | 2.29 | 2.15 | 126.06 |
|  | Stiripentol | 1.23 | 1.08 | 1.39 | <0.001 | 1.21 | 1.07 | 6.87 |
|  | Sultiame | 1.97 | 1.72 | 2.22 | <0.001 | 1.83 | 1.62 | 29.55 |
|  | Valproic acid and Sodium Valproate | 1.30 | 1.28 | 1.32 | <0.001 | 1.27 | 1.25 | 678.52 |
|  | Zonisamide | 1.70 | 1.63 | 1.77 | <0.001 | 1.61 | 1.55 | 225.02 |
| **Fatal** | Clonazepam | 2.56 | 2.54 | 2.59 | <0.001 | 2.43 | 2.40 | 4,474.94 |
|  | Ethotoin | 5.82 | 5.05 | 6.59 | <0.001 | 4.90 | 4.28 | 25.85 |
|  | Fosphenytoin | 3.15 | 3.01 | 3.29 | <0.001 | 2.91 | 2.78 | 277.10 |
|  | Gabapentin | 2.03 | 2.00 | 2.06 | <0.001 | 1.96 | 1.93 | 2,710.83 |
|  | Methylphenobarbital | 5.03 | 4.85 | 5.21 | <0.001 | 4.35 | 4.20 | 390.60 |
|  | Phenobarbital | 1.99 | 1.93 | 2.05 | <0.001 | 1.92 | 1.87 | 592.98 |
|  | Phenytoin | 1.47 | 1.42 | 1.51 | <0.001 | 1.44 | 1.40 | 278.49 |
|  | Primidone | 1.38 | 1.22 | 1.54 | <0.001 | 1.36 | 1.21 | 16.06 |
|  | Trimethadione | 5.64 | 5.04 | 6.25 | <0.001 | 4.78 | 4.29 | 40.43 |
|  | Vigabatrin | 1.78 | 1.71 | 1.86 | <0.001 | 1.73 | 1.66 | 257.57 |

**B** Outcome criterion with negative association of ASMs

| **Criterion** | **ASM** | **ROR** | **Lower 95%CI** | **Upper 95%CI** | **p-value** | **PRR** | **Lower 95%CI** | **Chi-square value** |
| --- | --- | --- | --- | --- | --- | --- | --- | --- |
| **Not Recovered/Not Resolved** | Barbexaclone | 0.54 | 0.48 | 0.59 | <0.001 | 0.57 | 0.52 | 486.22 |
|  | Carbamazepine | 0.53 | 0.51 | 0.56 | <0.001 | 0.57 | 0.55 | 2,361.97 |
|  | Clobazam | 0.79 | 0.74 | 0.85 | <0.001 | 0.82 | 0.77 | 67.50 |
|  | Fosphenytoin | 0.40 | 0.21 | 0.60 | <0.001 | 0.44 | 0.26 | 88.99 |
|  | Lamotrigine | 0.56 | 0.54 | 0.59 | <0.001 | 0.60 | 0.57 | 2,112.44 |
|  | Levetiracetam | 0.88 | 0.86 | 0.90 | <0.001 | 0.89 | 0.87 | 144.26 |
|  | Methylphenobarbital | 0.41 | 0.13 | 0.70 | 0.0047 | 0.45 | 0.18 | 39.47 |
|  | Oxcarbazepine | 0.79 | 0.75 | 0.83 | <0.001 | 0.81 | 0.78 | 128.72 |
|  | Phenobarbital | 0.53 | 0.47 | 0.58 | <0.001 | 0.56 | 0.51 | 519.12 |
|  | Phenytoin | 0.66 | 0.62 | 0.69 | <0.001 | 0.69 | 0.66 | 536.61 |
|  | Primidone | 0.75 | 0.63 | 0.87 | <0.001 | 0.77 | 0.67 | 23.38 |
|  | Retigabine | 0.75 | 0.57 | 0.92 | <0.001 | 0.77 | 0.61 | 10.63 |
|  | Rufinamide | 0.77 | 0.56 | 0.98 | <0.001 | 0.80 | 0.61 | 5.81 |
|  | Stiripentol | 0.29 | 0.07 | 0.52 | 0.01 | 0.32 | 0.11 | 126.04 |
|  | Tiagabine | 0.59 | 0.40 | 0.78 | <0.001 | 0.62 | 0.45 | 30.45 |
|  | Valproic acid and Sodium Valproate | 0.71 | 0.69 | 0.73 | <0.001 | 0.74 | 0.72 | 1,319.08 |
|  | Vigabatrin | 0.51 | 0.44 | 0.58 | <0.001 | 0.55 | 0.48 | 376.07 |
|  | Zonisamide | 0.91 | 0.84 | 0.98 | <0.001 | 0.92 | 0.86 | 6.95 |
| **Recovered/Resolved** | Brivaracetam | 0.78 | 0.72 | 0.83 | <0.001 | 0.81 | 0.77 | 76.66 |
|  | Clonazepam | 0.96 | 0.94 | 0.98 | <0.001 | 0.97 | 0.95 | 15.67 |
|  | Felbamate | 0.75 | 0.54 | 0.95 | <0.001 | 0.79 | 0.62 | 7.62 |
|  | Fenfluramine | 0.79 | 0.64 | 0.95 | <0.001 | 0.83 | 0.70 | 9.00 |
|  | Gabapentin | 0.69 | 0.67 | 0.71 | <0.001 | 0.74 | 0.72 | 1,616.15 |
|  | Lamotrigine | 0.88 | 0.86 | 0.90 | <0.001 | 0.90 | 0.89 | 207.50 |
|  | Pregabalin | 0.72 | 0.71 | 0.74 | <0.001 | 0.77 | 0.76 | 2,771.86 |
|  | Vigabatrin | 0.68 | 0.63 | 0.73 | <0.001 | 0.73 | 0.68 | 236.03 |
| **Recovered/Resolved with Sequelae** | Cannabidiol | 0.63 | 0.31 | 0.96 | <0.001 | 0.63 | 0.31 | 7.83 |
|  | Clonazepam | 0.72 | 0.59 | 0.85 | <0.001 | 0.72 | 0.59 | 23.60 |
|  | Gabapentin | 0.86 | 0.75 | 0.96 | <0.001 | 0.86 | 0.75 | 8.09 |
|  | Lacosamide | 0.78 | 0.60 | 0.96 | <0.001 | 0.78 | 0.60 | 7.42 |
|  | Pregabalin | 0.90 | 0.83 | 0.97 | <0.001 | 0.90 | 0.83 | 9.61 |
|  | Vigabatrin | 0.67 | 0.35 | 0.99 | <0.001 | 0.67 | 0.35 | 6.07 |
| **Recovering/Resolving** | Brivaracetam | 0.71 | 0.62 | 0.80 | <0.001 | 0.73 | 0.64 | 53.19 |
|  | Cannabidiol | 0.78 | 0.71 | 0.86 | <0.001 | 0.80 | 0.73 | 42.49 |
|  | Eslicarbazepine | 0.86 | 0.75 | 0.96 | <0.001 | 0.87 | 0.77 | 8.40 |
|  | Gabapentin | 0.76 | 0.73 | 0.79 | <0.001 | 0.77 | 0.75 | 374.50 |
|  | Lacosamide | 0.84 | 0.79 | 0.88 | <0.001 | 0.85 | 0.81 | 62.20 |
|  | Pregabalin | 0.70 | 0.68 | 0.72 | <0.001 | 0.72 | 0.70 | 1,335.30 |
|  | Tiagabine | 0.57 | 0.32 | 0.82 | <0.001 | 0.59 | 0.35 | 19.39 |
|  | Vigabatrin | 0.84 | 0.76 | 0.91 | <0.001 | 0.85 | 0.78 | 23.01 |
| **Fatal** | Brivaracetam | 0.25 | 0.04 | 0.46 | 0.02 | 0.26 | 0.05 | 194.94 |
|  | Carbamazepine | 0.82 | 0.78 | 0.86 | <0.001 | 0.83 | 0.79 | 96.87 |
|  | Cenobamate | 0.42 | 0.05 | 0.80 | 0.03 | 0.43 | 0.06 | 21.76 |
|  | Clobazam | 0.82 | 0.72 | 0.91 | <0.001 | 0.82 | 0.73 | 16.77 |
|  | Eslicarbazepine | 0.30 | 0.07 | 0.54 | 0.01 | 0.31 | 0.08 | 111.28 |
|  | Lacosamide | 0.79 | 0.73 | 0.85 | <0.001 | 0.80 | 0.74 | 54.26 |
|  | Lamotrigine | 0.72 | 0.68 | 0.76 | <0.001 | 0.73 | 0.69 | 252.69 |
|  | Levetiracetam | 0.83 | 0.79 | 0.86 | <0.001 | 0.83 | 0.79 | 95.57 |
|  | Oxcarbazepine | 0.77 | 0.70 | 0.84 | <0.001 | 0.78 | 0.71 | 49.08 |
|  | Perampanel | 0.40 | 0.19 | 0.60 | <0.001 | 0.40 | 0.21 | 88.23 |
|  | Pregabalin | 0.55 | 0.52 | 0.58 | <0.001 | 0.56 | 0.54 | 1,722.57 |
|  | Retigabine | 0.42 | 0.01 | 0.83 | 0.046 | 0.43 | 0.02 | 18.25 |
|  | Stiripentol | 0.50 | 0.18 | 0.83 | 0.003 | 0.51 | 0.19 | 17.85 |
|  | Tiagabine | 0.57 | 0.22 | 0.92 | 0.002 | 0.58 | 0.23 | 10.41 |
|  | Topiramate | 0.88 | 0.83 | 0.93 | <0.001 | 0.89 | 0.84 | 23.84 |
|  | Valproic acid and Sodium Valproate | 0.74 | 0.71 | 0.77 | <0.001 | 0.75 | 0.72 | 336.71 |

ASM: antiseizure medication, ROR: Reporting Odds Ratio, PRR: Proportional Reporting Ratio
