# Supplementary material for: Seriousness and outcomes of reported adverse drug reactions in old and new antiseizure medications: a pharmacovigilance study using EudraVigilance database
Source: Front Pharmacol. 2024 Jul 24;15:1411134. doi: 10.3389/fphar.2024.1411134 (PMC11307265; doi:10.3389/fphar.2024.1411134)
Supplement: Supplementary file 4 [file Table4.docx]

**Supplementary table 4** Comparison of outcomes of PTs by old ASMs and new ASMs

| **Outcome criterion** |  | **ROR** | **Lower 95%CI** | **Upper 95%CI** | **z statistic** | **p-value** | **PRR** | **Chi-square value** |
| --- | --- | --- | --- | --- | --- | --- | --- | --- |
| **Fatal** | Old ASMs | 1.37 | 1.35 | 1.39 | 133.67 | p<0.001 | 1.35 | 939.15 |
|  | *Male* | *0.94* | *0.92* | *0.97* | *66.35* | *p<0.001* | *0.95* | *16.36* |
|  | *Female* | *1.15* | *1.12* | *1.17* | *90.92* | *p<0.001* | *1.14* | *121.88* |
|  | New ASMs | 0.73 | 0.71 | 0.75 | 71.60 | p<0.001 | 0.74 | 939.15 |
|  | *Male* | *1.30* | *1.28* | *1.32* | *113.08* | *p<0.001* | *1.29* | *521.83* |
|  | *Female* | *0.64* | *0.61* | *0.66* | *57.79* | *p<0.001* | *0.65* | *1,707.60* |
| **Not recovered/Not resolved** | Old ASMs | 0.71 | 0.70 | 0.72 | 111.50 | p<0.001 | 0.74 | 2,924.76 |
|  | *Male* | *0.36* | *0.33* | *0.38* | *31.63* | *p<0.001* | *0.39* | *9,213.21* |
|  | *Female* | *0.88* | *0.86* | *0.89* | *112.05* | *p<0.001* | *0.89* | *281.13* |
|  | New ASMs | 1.41 | 1.40 | 1.42 | 221.41 | p<0.001 | 1.35 | 2,924.76 |
|  | *Male* | *1.03* | *1.02* | *1.05* | *147.86* | *p<0.001* | *1.03* | *23.00* |
|  | *Female* | *1.50* | *1.49* | *1.51* | *257.32* | *p<0.001* | *1.42* | *4,903.69* |
| **Recovered/Resolved** | Old ASMs | 1.32 | 1.31 | 1.33 | 266.83 | p<0.001 | 1.25 | 3,217.77 |
|  | *Male* | *0.67* | *0.66* | *0.69* | *91.33* | *p<0.001* | *0.72* | *2,971.72* |
|  | *Female* | *1.30* | *1.29* | *1.31* | *215.22* | *p<0.001* | *1.23* | *1,883.17* |
|  | New ASMs | 0.75 | 0.75 | 0.76 | 152.08 | p<0.001 | 0.80 | 3,217.77 |
|  | *Male* | *1.07* | *1.06* | *1.08* | *184.07* | *p<0.001* | *1.05* | *128.91* |
|  | *Female* | *0.91* | *0.90* | *0.92* | *181.91* | *p<0.001* | *0.92* | *387.22* |
| **Recovered/Resolved with sequelae** | Old ASMs | 1.18 | 1.12 | 1.24 | 40.07 | p<0.001 | 1.18 | 32.26 |
|  | *Male* | *0.62* | *0.53* | *0.72* | *13.12* | *p<0.001* | *0.62* | *100.89* |
|  | *Female* | *1.22* | *1.15* | *1.29* | *34.45* | *p<0.001* | *1.22* | *31.43* |
|  | New ASMs | 0.85 | 0.79 | 0.90 | 28.67 | p<0.001 | 0.85 | 32.26 |
|  | *Male* | *1.14* | *1.07* | *1.20* | *33.70* | *p<0.001* | *1.14* | *14.53* |
|  | *Female* | *0.93* | *0.87* | *0.99* | *31.30* | *p<0.001* | *0.93* | *5.95* |
| **Recovering/Resolving** | Old ASMs | 1.51 | 1.49 | 1.52 | 209.29 | p<0.001 | 1.46 | 3,282.49 |
|  | *Male* | *0.84* | *0.82* | *0.86* | *81.03* | *p<0.001* | *0.85* | *274.47* |
|  | *Female* | *1.37* | *1.35* | *1.38* | *158.66* | *p<0.001* | *1.33* | *1,322.44* |
|  | New ASMs | 0.66 | 0.65 | 0.68 | 92.10 | p<0.001 | 0.69 | 3,282.49 |
|  | *Male* | *1.01* | *1.00* | *1.03* | *117.49* | *p<0.001* | *1.01* | *2.01* |
|  | *Female* | *0.82* | *0.81* | *0.84* | *110.60* | *p<0.001* | *0.84* | *683.07* |

ASM: antiseizure medication, ROR: Reporting Odds Ratio, PRR: Proportional Reporting Ratio
